# Tonicity-responsive enhancer-binding protein promotes diabetic neuroinflammation and cognitive impairment via upregulation of lipocalin-2

**DOI:** 10.1186/s12974-021-02331-8

**Published:** 2021-11-29

**Authors:** Eun Ae Jeong, Jaewoong Lee, Hyun Joo Shin, Jong Youl Lee, Kyung Eun Kim, Hyeong Seok An, Deok Ryong Kim, Kyu Yeong Choi, Kun Ho Lee, Gu Seob Roh

**Affiliations:** 1 Department of Anatomy and Convergence Medical Science, Bio Anti-Aging Medical Research Center, Institute of Health Sciences, College of Medicine, Gyeongsang National University, Jinju, 52727 Republic of Korea; 2 Department of Biochemistry, Institute of Health Sciences, College of Medicine, Gyeongsang National University, Jinju, 52727 Republic of Korea; 3 Gwangju Alzheimer’s Disease and Related Dementia Cohort Research Center, Chosun University, Gwangju, 61452 Republic of Korea; 4 Department of Biomedical Science, Chosun University, Gwangju, 61452 Republic of Korea; 5 Aging Neuroscience Research Group, Korea Brain Research Institute, Daegu, 41062 Republic of Korea

**Keywords:** TonEBP, Lipocalin-2, Neuroinflammation, Hippocampus, Diabetes

## Abstract

**Background:**

Diabetic individuals have increased circulating inflammatory mediators which are implicated as underlying causes of neuroinflammation and memory deficits. Tonicity-responsive enhancer-binding protein (TonEBP) promotes diabetic neuroinflammation. However, the precise role of TonEBP in the diabetic brain is not fully understood.

**Methods:**

We employed a high-fat diet (HFD)-only fed mice or HFD/streptozotocin (STZ)-treated mice in our diabetic mouse models. Circulating TonEBP and lipocalin-2 (LCN2) levels were measured in type 2 diabetic subjects. TonEBP haploinsufficient mice were used to investigate the role of TonEBP in HFD/STZ-induced diabetic mice. In addition, RAW 264.7 macrophages were given a lipopolysaccharide (LPS)/high glucose (HG) treatment. Using a siRNA, we examined the effects of TonEBP knockdown on RAW264 cell’ medium/HG-treated mouse hippocampal HT22 cells.

**Results:**

Circulating TonEBP and LCN2 levels were higher in experimental diabetic mice or type 2 diabetic patients with cognitive impairment. TonEBP haploinsufficiency ameliorated the diabetic phenotypes including adipose tissue macrophage infiltrations, neuroinflammation, blood–brain barrier leakage, and memory deficits. Systemic and hippocampal LCN2 proteins were reduced in diabetic mice by TonEBP haploinsufficiency. TonEBP (+ / −) mice had a reduction of hippocampal heme oxygenase-1 (HO-1) expression compared to diabetic wild-type mice. In particular, we found that TonEBP bound to the LCN2 promoter in the diabetic hippocampus, and this binding was abolished by TonEBP haploinsufficiency. Furthermore, TonEBP knockdown attenuated LCN2 expression in lipopolysaccharide/high glucose-treated mouse hippocampal HT22 cells.

**Conclusions:**

These findings indicate that TonEBP may promote neuroinflammation and cognitive impairment via upregulation of LCN2 in diabetic mice.

**Supplementary Information:**

The online version contains supplementary material available at 10.1186/s12974-021-02331-8.

## Introduction

Type 2 diabetes mellitus (T2DM) is accompanied by chronic low-grade inflammation, which in combination with hyperglycemia, leads to severe co-morbidities such as cardiovascular and neurodegenerative diseases [1, 2]. Adipocytokines from adipose tissue are important sources of inflammation in obesity and T2DM [3, 4]. These active mediators and proinflammatory cytokines contribute to insulin resistance, oxidative stress, neuroinflammation, and memory deficits [5–8]. In diabetes, the hippocampus is easily impacted by the active mediators TNF-α and IL-6 through the breakdown of the blood–brain barrier (BBB) [7–9]. These evidences suggest that the increased adipocytokines under chronic diabetic conditions could be functionally associated with other inflammatory mediators in the diabetic brain, but the underlying molecular mechanisms responsible for diabetic encephalopathy remain unclear.

Lipocalin-2 (LCN2) is expressed in adipose tissue, liver, and brain [4, 10–13]. LCN2 is particularly associated with several brain injuries such as ischemic stroke, diabetes, dementia, and encephalomyelitis [13–16]. Although LCN2 is originally isolated from neutrophil granules, it is functionally connected to inflammation, because the LCN2 gene has a nuclear factor-κB (NF-κB) binding site in its promoter region [17, 18]. Nevertheless, LCN2 has been shown to have both protective and pathogenic roles in organ damage with rodents or humans, including hepatic injury, sepsis, obesity, and diabetes [19–22]. Therefore, further functional regulation of LCN2 in the progression of diabetic encephalopathy is necessary.

Tonicity-responsive enhancer-binding protein (TonEBP) is associated with NF-κBp65-mediated inflammation in macrophages [23, 24], and TonEBP-mediated pathological osmoadaptation is involved in acute systemic hypertonicity and neurological disease [25, 26]. Furthermore, TonEBP haploinsufficiency has a protective role in neuroinflammatory diseases including seizures, ischemic brain injury, and diabetes [24, 26, 27]. Based on its neuroinflammatory role through crosstalk between macrophage and brain under diabetic conditions, we hypothesized that TonEBP might be crucial for the development of neuroinflammation in the diabetic brain.

As TonEBP and LCN2 have several roles in common for chronic low-grade inflammation in diabetes, functional connections between them in macrophage-mediated neuroinflammation would be expected in the diabetic brain. We previously showed that TonEBP has an inflammatory role in the diabetic brain [24]. However, the exact mechanisms of TonEBP-mediated diabetic neuroinflammation and memory deficits have not been fully studied. Here, we demonstrate that TonEBP transcriptionally regulates intracellular levels of LCN2 in macrophages and the hippocampus under chronic diabetic conditions. In addition, we show that TonEBP has direct binding to LCN2's regulatory-element region using a ChIP assay and siRNA-mediated silencing in RAW 264.7 macrophages and HT22 neuronal cells. Thus, a better understanding of diabetic encephalopathy due to systemic inflammation is necessary for the development of new therapeutic strategies.

## Materials and methods

### Animals

TonEBP heterozygote mice were obtained from Dr. Kwon (Ulsan National Institute of Science and Technology). The TonEBP (+ / −) mice were crossed back to the C57BL/6 J (The Central Laboratory Animal Inc. Seoul, South Korea) to produce TonEBP (+ / −) mice and their TonEBP (+ / +) littermates. In particular, as homozygous deletion of TonEBP results in significant atrophy of the renal medulla, TonEBP (+ / −) mice were used to study the role of TonEBP in this diabetic study [28, 29]. All experiments were performed in accordance with the National Institutes of Health Guidelines on the Use of Laboratory Animals. The University Animal Care Committee for Animal Research at Gyeongsang National University (GNU) approved the study protocol (GNU-160530-M0025). Male mice were individually housed with an alternating 12 h light/dark cycle.

### Human samples

The study protocol was approved by the Institutional Review Board of Chosun University Hospital, Gwangju, Korea (CHOSUN 2013-12-018-070). A total of controls, individuals with diabetes, and diabetic patients with mild cognitive impairment (MCI) were recruited from a pool of individuals registered in Gwangju Alzheimer’s Disease and Related Dementia Cohort Research Center, Chosun University, Gwangju, South Korea from January 2014 to April 2016. Participants provided fasting blood samples for measures of metabolic parameters at Samkwang Medical Laboratories (Gwangju, South Kora). A comprehensive neuropsychological assessment was performed using the Seoul Neuropsychological Screening Battery (SNSB), which covers five major cognitive domains, memory, attention, language, visuospatial function, and frontal/executive function domains [30]. Global cognition was assessed using the Korean version of the Mini-Mental State Examination (K-MMSE) [31].

### Diabetic mouse models

For HFD-fed or HFD/STZ-treated diabetic mice, male mice were fed for 20 weeks with either a HFD (60%, Research Diets Inc, New Brunswick, NJ, USA) or normal diet (ND) chow. At 16 weeks on a HFD, mice were injected with 100 mg/kg of STZ (ChromaDex Inc, Irvine, CA, USA) or vehicle. One month after STZ injection, mice were just before sacrifice at 24 weeks of age.

### Glucose-tolerance testing (GTT) and insulin-tolerance testing (ITT)

GTT and ITT were performed as previously described [8]. Briefly, D-glucose (2 g/kg, Sigma-Aldrich, St. Louis, MO, USA) or insulin (0.75 U/kg, Humulin-R, Eli Lilly, Indianapolis, IN, USA) was injected intraperitoneally and blood samples were taken before and after the injections using an Accu-Chek glucometer (Roche Diagnostics GmbH, Mannheim, Germany).

### Metabolic parameters

After overnight fasting, mice were anesthetized with zoletil (5 mg/kg, Virbac Laboratories, Carros, France). Blood samples were taken from the left ventricle and centrifuged. Serum glucose, aspartate aminotransferase (AST) and alanine aminotransferase (ALT) levels were determined at the Green Cross Reference Laboratory (Youngin-si, South Korea). For the hepatic triglyceride (TG) colorimetric assay, frozen livers were used to determine TG levels. TG concentrations were measured using a TG colorimetric assay kit (Cayman Chemical Company, Ann Arbor, MI, USA) according to the manufacturer’s protocol. An EchoMRI (Whole Body Composition Analyzer, Houston, TX, USA) was performed on animals to quantify body fat and lean mass.

### Enzyme-linked immunosorbent assay (ELISA)

Serum insulin and C-peptide were measured using a mouse insulin (Shibayagi Co., Gunma, Japan) enzyme-linked immunosorbent assay (ELISA) kit. Serum LCN2 and TonEBP were measured using mouse TonEBP (EIAab Science Co., Ltd, Wuhan, China), human TonEBP (MyBioSource, Inc., San Diego, CA, USA), mouse LCN2 (R&D Systems, Minneapolis, MN, USA), and human LCN2 (R&D Systems) according to manufacturer’s protocols.

### Hematoxylin and eosin (H&E), oil red o, and nile red staining

To histologically score non-alcoholic fatty liver disease (NAFLD) activity, paraffin-embedded liver sections were used. The NAFLD activity score was quantified by summing the scores for steatosis (0–3), lobular inflammation (0–2), and hepatocellular ballooning (0–2). To determine hepatic lipid accumulation, frozen liver Sections (5 μm) were stained with 0.5% Oil Red O (Sigma-Aldrich) or Nile Red (Sigma-Aldrich) for 10 min, washed, and counterstained with Mayer’s hematoxylin (Sigma-Aldrich) for 45 s. Crown like structures (CLSs) in adipose tissue were identified using H&E staining. Tissue sections were visualized under a BX51 light microscope (Olympus, Tokyo, Japan) and digital images were captured and documented.

### Immunohistochemistry

Deparaffinized epididymal fat pads and frozen-brain sections were incubated overnight at 4 °C in a humidified chamber with primary antibodies (Additional file 1: Table S1) diluted in blocking serum. After washing, sections were incubated in avidin–biotin–peroxidase complex solution (Vector Laboratories, Burlingame, CA, USA). Sections were developed with 0.05% diaminobenzidine (DAB, Sigma-Aldrich) containing 0.05% H_2_O_2_ and were dehydrated through graded alcohols, cleared in xylene, and coverslipped with Permount (Sigma-Aldrich). Sections were visualized using BX51 light microscopy (Olympus). For the measurement of the intensity of immunostained TonEBP from brain sections, three fields (200 × 200 µm^2^) were randomly selected from each section (*n* = 3–4 per group).

### Immunofluorescence

Deparaffinized pancreatic and epididymal fat pads, frozen-brain sections, or cells were incubated overnight with primary antibodies at 4 °C (Additional file 1: Table S1). After washing, sections or cells were incubated with AlexaFluor 488-, 594-, and 680-conjugated donkey secondary antibodies (Invitrogen). Nuclei were stained with DAPI (1:20,000, Invitrogen). In addition, we performed double-immunofluorescence staining for insulin and terminal deoxynucleotidyl transferase dUTP nick-end labeling (TUNEL) to measure the degree of insulin-positive pancreatic β-cells in pancreatic sections using an in-situ cell death detection kit (Roche Molecular Biochemicals, Mannheim, Germany) according to the manufacturer's instructions. In particular, we measured the number of TUNEL-positive cells within Langerhans islets area. Fluorescence was visualized using a confocal microscope (FV-1000, Olympus). For the measurement of the intensity of immunostained protein from brain sections, three fields (200 × 200 µm^2^) were randomly selected from each section (*n* = 3–4 per group).

### Cell culture

The macrophage cell line, RAW 264.7, and the mouse hippocampal cell line, HT22 (ATTC, Rockville, MD, USA), were cultured in Dulbecco's modified Eagle medium (Gibco, Grand Island, USA) supplemented with 10% fetal bovine serum (Gibco), 1% penicillin/streptomycin (Gibco) at 37 °C in a 5% CO_2_ humidified incubator. RAW 264.7 cells were plated at a density of 3 × 10^5^ cells per 60 mm dish. The cells were rinsed with fresh medium and stimulated at the indicated timepoints with lipopolysaccharide (LPS; 100 ng/ml). For inflammatory and hyperglycemic conditions, RAW 264.7 cells were co-treated with LPS and 25 mM glucose for 24 h. For conditions of an inflammatory BBB-crossing response, after incubation with 125 mM glucose for 24 h, HT22 cells were treated for 24 h with LPS/HG-treated RAW 264.7 cell medium (LTM).

### Transfection of small interfering RNA

Small interfering RNA (siRNA) targeting TonEBP and a scrambled-control (Bioneer Corp., Daejon, South Korea) and LCN2 (Santa Cruz Biotechnology, Santa Cruz, CA, USA) were purchased. Cells were transfected with TonEBP siRNA, LCN2 siRNA, control scrambled siRNA using lipofectamine RNAiMAX (Invitrogen, Carlsbad, CA, USA) following the manufacturer’s instructions.

### Mitochondrial superoxide (MitoSOX) assay

The MitoSOX assay was carried out using MitoSOX™ Red mitochondrial superoxide indicator (Invitrogen) according to the manufacturer’s instructions. Briefly, HT22 cells were incubated with 125 mM glucose for 24 h before being treated with LTM for 24 h. After 48 h, cells were incubated with 5 μM MitoSOX at 37 °C for 10 min. The cells were then fixed with 4% paraformaldehyde in PBS for 10 min. After washing three times with 0.1 M PBS, nuclei were stained with DAPI (1:20,000, Invitrogen) for 10 min. Fluorescence was visualized using a confocal microscope (FV-1000, Olympus, Tokyo, Japan).

### RNA extraction and real-time PCR

Total RNA was extracted from epididymal fat pads and cells using TRIzol reagent (Invitrogen) and reverse-transcribed using the RevertAid™ First-Strand cDNA Synthesis a Kit (Fermentas Inc., Hanover, MD, USA). RT-PCR was performed using the ABI Prism 7000 Sequence Detection System (Applied Biosystems, Foster City, CA, USA). PCR amplifications were performed using an SYBR Green I qPCR kit (TaKaRa, Shiga, Japan) with specific primers (Additional file 1: Table S2). Relative quantifications were calculated using the ∆∆Ct formula. Relative mRNA expression was expressed as fold-change relative to a calibrator sample.

### Western blot analyses

Frozen epididymal fat pads and hippocampi were homogenized in T-PER® Tissue Protein Extraction Reagent (Thermofisher Scientific, Waltham, MA, USA) for protein extraction. For cytosolic and nuclear fractions, we used the NE-PER Nuclear and Cytoplasmic Extraction Kit (Pierce, Rockford, IL, USA). Protein concentrations were determined using a bicinchoninic acid assay (Pierce), and samples were stored at − 80 °C until use. Western blot analyses were performed using standard methods, and proteins were immunoblotted with primary antibodies (Additional file 1: Table S1). The membranes were probed with each internal control and visualized using an enhanced chemiluminescence substrate (Pierce). The Multi-Gauge V 3.0 image analysis program (Fujifilm, Tokyo, Japan) was used to measure band densitometry.

### Chromatin immunoprecipitation (ChIP) assay

The ChIP assay was carried out using the High-Sensitivity ChIP kit (Abcam, Paris, France) following the manufacturer’s directions. After lysis and sonication, samples were incubated overnight at 4 °C with anti-TonEBP (Thermofisher Scientific), anti-NF-κBp65 (Cell signaling), or Non-Immune IgG (from the High-Sensitivity ChIP kit) as a negative control. Immune complexes were subjected to reverse crosslinking, release, and purification of DNA using DNA Release buffer, and RNase A (from the High-Sensitivity ChIP kit). DNA was then subjected to RT-PCR using the primers listed in Additional file 1: Table S3. Immunoprecipitated DNA from each sample was normalized to its respective chromatin input.

### Morris water maze test

Morris water maze test was performed as previously described [8]. All mice were subjected to four trials per day for four consecutive days. The escape latency to find the platform was recorded by a video-tracking program (Noldus EthoVision XT7, Noldus Information Technology, Netherlands). On the day of testing, the platform was removed, and time spent in the target quadrant, where the platform had been located during training, was analyzed.

### Statistics

Statistical analyses were performed using PRISM 7.0 (GraphPad Software Inc., San Diego, CA, USA). All values are expressed as mean ± SEM. A *p* value less than 0.05 was considered statistically significant.

## Results

### Experimental diabetic mice exhibit diabetic phenotypes with memory deficits

**Fig. 1:**
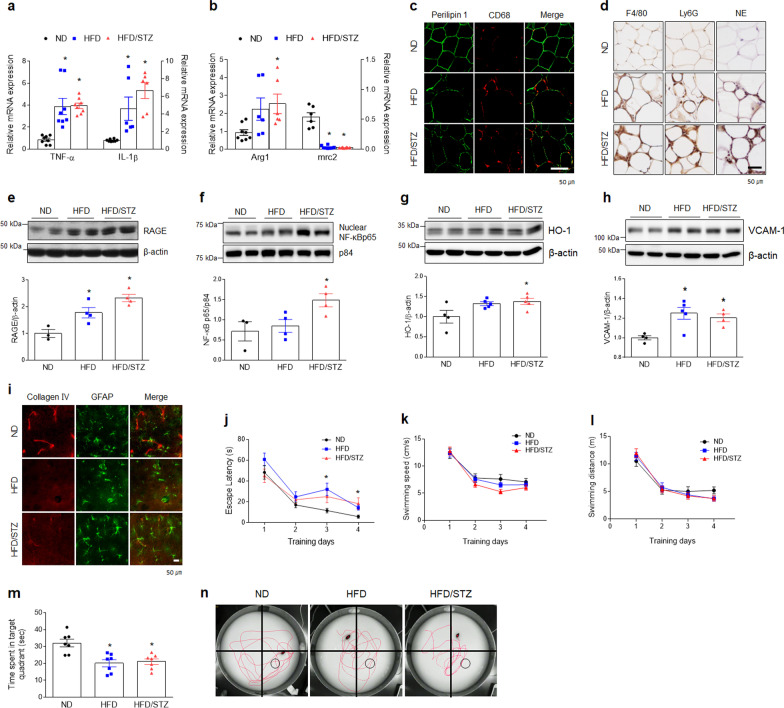
Systemic inflammation and neuroinflammation are associated with HFD- or HFD/STZ-induced diabetic mice with memory deficits.** a**, **b** Expressions of mRNA encoding TNF-α (*F* = 14.85, *p* < 0.001), IL-1β (*F* = 12.71, *p* < 0.001), Arg1 (F = 4.039, *p* = 0.037), and mrc2 (*F* = 66.75, *p* < 0.001) in adipose tissue were measured by quantitative RT-PCR (*n* = 6–8). **c** Representative immunofluorescence staining for perilipin 1 and CD68 in adipose tissue sections. CD68-positive macrophages are arranged in crown-like structures (CLSs) around necrotic perilipin1-free adipocytes in HFD-fed mice with or without STZ (*n* = 3–4). **d** Representative images showing CLSs in adipose tissue. Immunohistochemical staining for macrophage-specific F4/80, neutrophil-specific Ly6G, and NE are colored brown. All sections were counterstained with hematoxylin (*n* = 3–4). **e–h** Western blots and quantified hippocampal RAGE **e** (*F* = 14.78, *p* = 0.002), nuclear NF-κBp65 **f** (*F* = 5.173, *p* = 0.036), HO-1 **g** (*F* = 4.304, *p* = 0.042), and VCAM-1 **h** (*F* = 7.729, *p* = 0.009). To normalize total and nuclear protein levels, β-actin and p84 were used, respectively. Data (*n* = 3–5) are shown as mean ± SEM. **i** Representative immunofluorescence staining for collagen IV (vascular marker) and GFAP (a marker for astrocyte) in the hippocampal CA1 regions (*n* = 3–4). **j**–**n** Escape latency **j** (*F* = 3.261, *p* = 0.045), Swimming speed **k** (*F* = 0.72, *p* = 0.691), and Swimming distance **l** (*F* = 1.104, *p* = 0.359) over four days of Morris water maze training, Time in the target platform quadrant **m** (*n* = 7, *F* = 10.76, *p* < 0.001), **n** Representative swim paths during the probe trial. Circle boxes indicate the location of the hidden platform. The indicated *p* values represent a one-way ANOVA followed by Tukey’s post-hoc test. *p < 0.05, versus ND. ^†^*p* < 0.05, versus HFD

We established experimental diabetic mouse models using HFD-only fed mice or HFD/STZ-treated mice (Additional file 1: Fig. S1a). Both HFD-only fed and HFD/STZ-treated mice exhibited diabetic phenotypes characterized by weight gain and NAFLD compared to ND-fed mice (Additional file 1: Fig. S1b–h). In particular, HFD/STZ-induced diabetic mice presented with higher glucose levels and partial loss of functional β-cell mass in the pancreas compared to HFD-only fed mice (Additional file 1: Fig. S2). Two experimental diabetic mouse models had higher mRNA expression levels of M1 polarization genes *(TNF-α* and *IL-1β*) and lower mRNA expression for an M2 gene (*mrc2*) in adipose tissues (Fig. 1a, b). Many cells positive for macrophage marker (F4/80 and CD68) and neutrophil markers [neutrophil elastase (NE) and Ly6G], which were arranged in CLSs around necrotic perilipin1-free adipocytes were observed in epididymal adipose tissues of two diabetic mouse models compared to ND-fed mice (Fig. 1c, d). In the hippocampus, the expressions of receptor for advanced glycation end products (RAGE), nuclear NF-κBp65, and heme oxygenase-1 (HO-1) were dramatically increased in HFD/STZ-treated diabetic mice compared to ND-fed mice (Fig. 1e–g). Furthermore, as vascular cell adhesion molecule 1 (VCAM1) expression increased and basement membrane collagen IV decreased in hippocampal CA1 regions, we suggest that BBB leakage occurred in two diabetic mouse brains compared to ND-fed mice (Fig. 1h, i). Two diabetic mouse models exhibited memory deficits compared to ND-fed mice despite no difference in swim speed and distance (Fig. 1j–n). Taken together, these findings indicate that diabetic encephalopathy is closely linked to M1 macrophage infiltration, neuroinflammation, BBB leakage, and cognitive impairment.

### Increased level of TonEBP is correlated with induction of LCN2 protein in diabetic mice

**Fig. 2:**
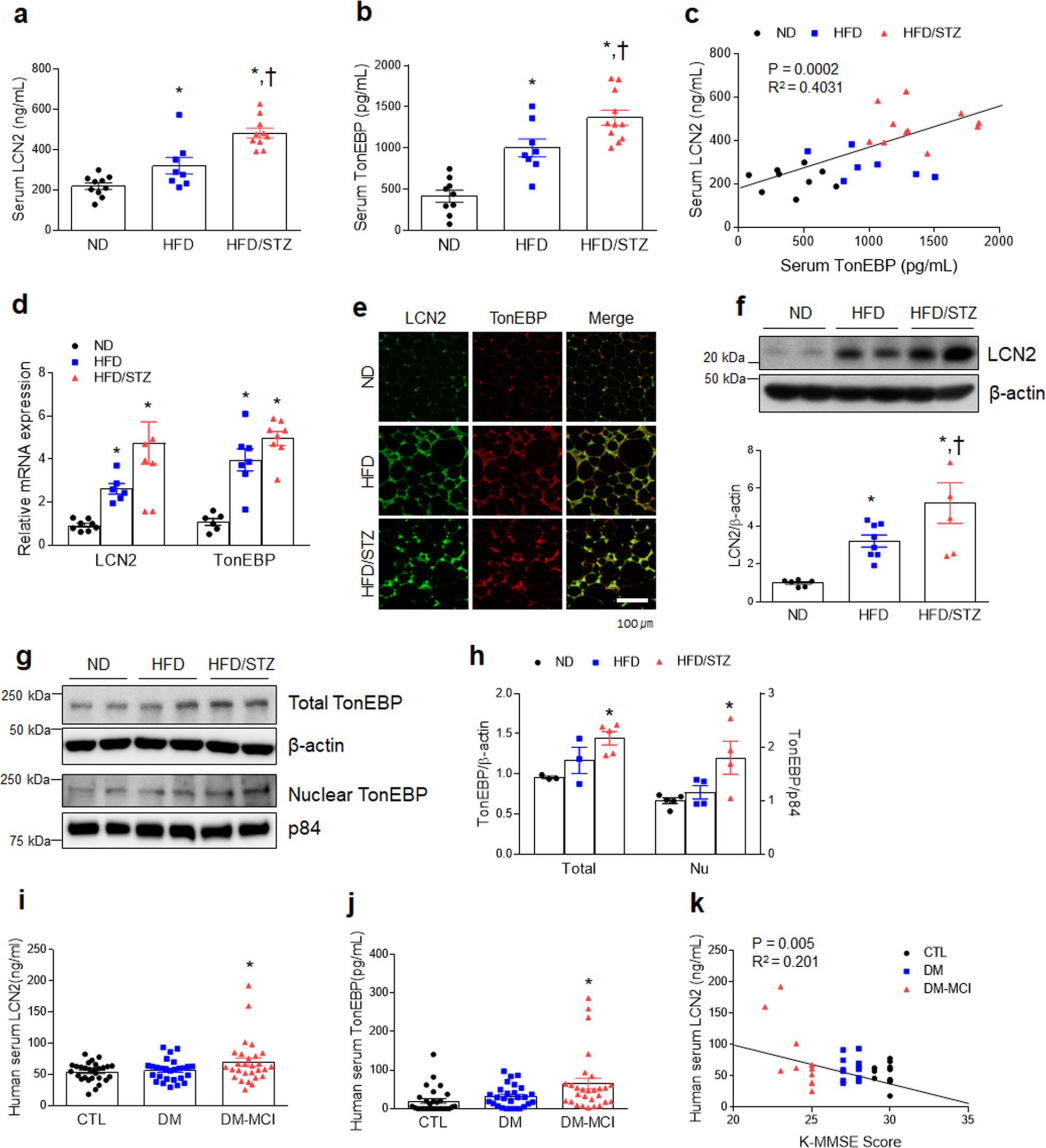
LCN2 and TonEBP are markedly increased in experimental diabetic mice and diabetic patients.** a**, **b** Serum LCN2 **a** (*F* = 21.43, *p* < 0.001) and TonEBP **b** (*F* = 28.9, *p* < 0.001) levels were measured using ELISAs (*n* = 8–11). **c** Correlation between serum LCN2 and TonEBP levels (*n* = 7–11). **d** Expression of LCN2 (*F* = 9.924, *p* < 0.001) and TonEBP (*F* = 25.6, *p* < 0.001) mRNA in adipose tissue (*n* = 6–8). **e** Representative immunofluorescence staining for TonEBP and LCN2 in adipose tissue sections (*n* = 3–4). **f** Western blots and quantified hippocampal expression of LCN2 (*n* = 5–8, *F* = 11.21, *p* < 0.001). Band intensities were normalized to β-actin. **g, h** Western blots and quantified hippocampal expression of total TonEBP (*n* = 3–5, *F* = 6.234, *p* = 0.023) and nuclear TonEBP (*n* = 4–5, *F* = 5.566, *p* = 0.024). To normalize the total and nuclear protein levels, β-actin and p84 were used, respectively. **i**, **j** Circulating LCN2 **i** (*F* = 3.391, *p* = 0.039) and TonEBP **j** (*F* = 4.527, *p* = 0.014) levels in control (CTL), diabetes (DM), and DM + mild cognitive impairment (MCI) subject samples were measured using ELISAs (*n* = 27–29). **k** Correlation between serum LCN2 and Korean Mini Mental State Examination (K-MMSE) scores (*n* = 12–13). Data are shown as mean ± SEM. *p < 0.05 versus CTL. The indicated *p* values represent a one-way ANOVA followed by Tukey’s post-hoc test. *p < 0.05, versus ND. ^†^*p* < 0.05, versus HFD

To investigate the relationship between LCN2 and TonEBP in diabetic mice, we first examined the expression profiles of LCN2 and TonEBP in serum, adipose tissue, and hippocampus. Notably, the serum levels of LCN2 and TonEBP were markedly elevated in HFD/STZ-treated diabetic mice compared to ND- or HFD-fed mice (Fig. 2a, b). Of particular interest, the circulating protein levels of LCN2 and TonEBP were positively correlated (*P* = 0.0002, *R*^2^ = 0.4031) (Fig. 2c). In addition to increased *LCN2* and *TonEBP* mRNA levels in adipose tissue of diabetic mice (Fig. 2d), TonEBP-positive cells were observed in LCN2-positive cells in adipose tissues of HFD-fed or HFD/STZ-treated mice (Fig. 2e). In addition to increased hippocampal LCN2 protein, total and nuclear TonEBP proteins significantly increased in the hippocampus of HFD/STZ-treated mice compared to HFD-fed mice (Fig. 2f–h). Indeed, nuclear TonEBP hippocampal neurons were increased in HFD/STZ-treated neurons compared to HFD-only fed mice (Additional file 1: Fig. S3a, b). LCN2-positive cells were observed together with neurons and glial fibrillary acidic protein (GFAP)-positive astrocytes (Additional file 1: Fig. S3c). Furthermore, these biologically significant findings in diabetic mice were validated in human patients with T2DM (Additional file 1: Table S4). The circulating levels of LCN2 and TonEBP were the highest in the diabetic subjects (HbA1C > 6.5) with MCI relative to normal subjects (Fig. 2i, j). Among diabetic patients with MCI, two diabetic subjects had the highest serum TonEBP and LCN2 levels as well as the lowest K-MMSE scores. Notably, LCN2 serum levels were inversely correlated with scores of the K-MMSE in diabetic patients with MCI (*R*^2^ = 0.201, *P* = 0.005) (Fig. 2k). Based on these findings, we speculate that TonEBP could regulate the induction of systemic and hippocampal LCN2 under diabetic conditions.

### TonEBP haploinsufficiency attenuates M1 macrophage and LCN2 protein in adipose tissue of HFD/STZ-treated diabetic mice

**Fig. 3:**
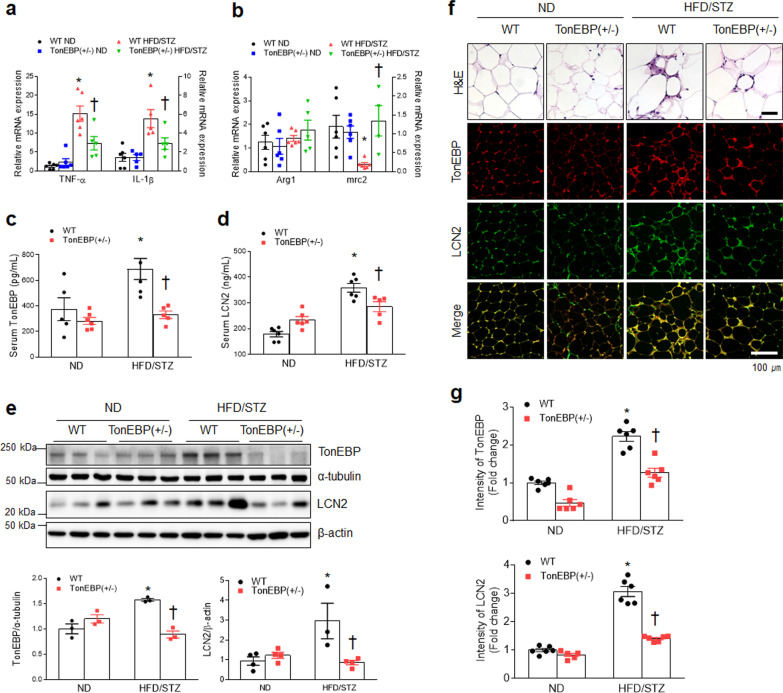
TonEBP haploinsufficiency attenuates systemic inflammation and memory deficits in HFD/STZ-induced diabetic mice.** a**, **b** Expressions of mRNAs encoding TNF-α (*F* = 10.11, *p* = 0.005), IL-1β (*F* = 4.594, *p* = 0.047), Arg1 (*F* = 0.7228, *p* = 0.406), and mrc2 (*F* = 7.333, *p* = 0.014) in adipose tissues were measured by quantitative RT-PCR (*n* = 4–6). **c, d** Circulating TonEBP **c** (*F* = 4.3, *p* = 0.053) and LCN2 **d** (*F* = 18.12, *p* < 0.001) levels were measured using ELISAs (*n* = 5–6).** e** Western blots and quantified adipose tissue expression of TonEBP (*F* = 35.91, *p* < 0.001) and LCN2 (*F* = 10.07, *p* = 0.009) (*n* = 3–4). **f** Representative H&E and immunostaining images for TonEBP and LCN2 (*n* = 3–4). **g** Quantification of TonEBP (F = 4.522, *p* = 0.046) or LCN2 (F = 57.99, *p* < 0.001) -positive cell counts in the images (*n* = 3–4). Data are shown as mean ± SEM. The indicated *p* values represent a two-way ANOVA followed by Tukey’s post-hoc test. *p < 0.05 versus WT ND. ^†^*p* < 0.05 versus WT HFD/STZ

We investigated the physiological role of LCN2 in HFD/STZ-treated diabetic TonEBP (+ /−) mice. Because complete loss of TonEBP function causes late gestational lethality under hyperosmotic conditions, TonEBP (+ / −) mice were used in this diabetic mouse model [28, 29]. Although TonEBP haploinsufficiency did not improve insulin resistance in diabetic mice, it reduced body weight and fat weight and improved NAFLD compared to diabetic WT mice (Additional file 1: Fig. S4). We also observed that increased M1 gene expression (*Tnf-α* and *Il-1β*) in adipose tissues of diabetic WT mice was decreased by TonEBP haploinsufficiency (Fig. 3a). In contrast, there was no significant change in M2 gene expressions (Fig. 3b). Consistent with changed serum levels of TonEBP and LCN2, increased level of TonEBP and LCN2 proteins in adipose tissues of diabetic WT mice was also attenuated by TonEBP haploinsufficiency (Fig. 3c–e). We found fewer CLSs in adipose tissue of diabetic TonEBP (+ / −) mice compared to that of diabetic WT mice, and the increased number of TonEBP- or LCN2-positive cells in diabetic WT mice was reduced in adipocytes of diabetic TonEBP (+ / −) mice (Fig. 3f, g). As expected, both fewer TonEBP and LCN2 proteins were co-localized in NE- and CD11b-positive neutrophils in adipose tissues of HFD/STZ-treated TonEBP (+ / −) mice compared to HFD/STZ-treated WT mice (Additional file 1: Fig. S5). These findings suggest that TonEBP could attenuate LCN2-positive inflammatory cells in diabetic mice.

### TonEBP haploinsufficiency inhibits BBB leakage, neuroinflammation, and memory deficits in HFD/STZ-treated diabetic mice

**Fig. 4:**
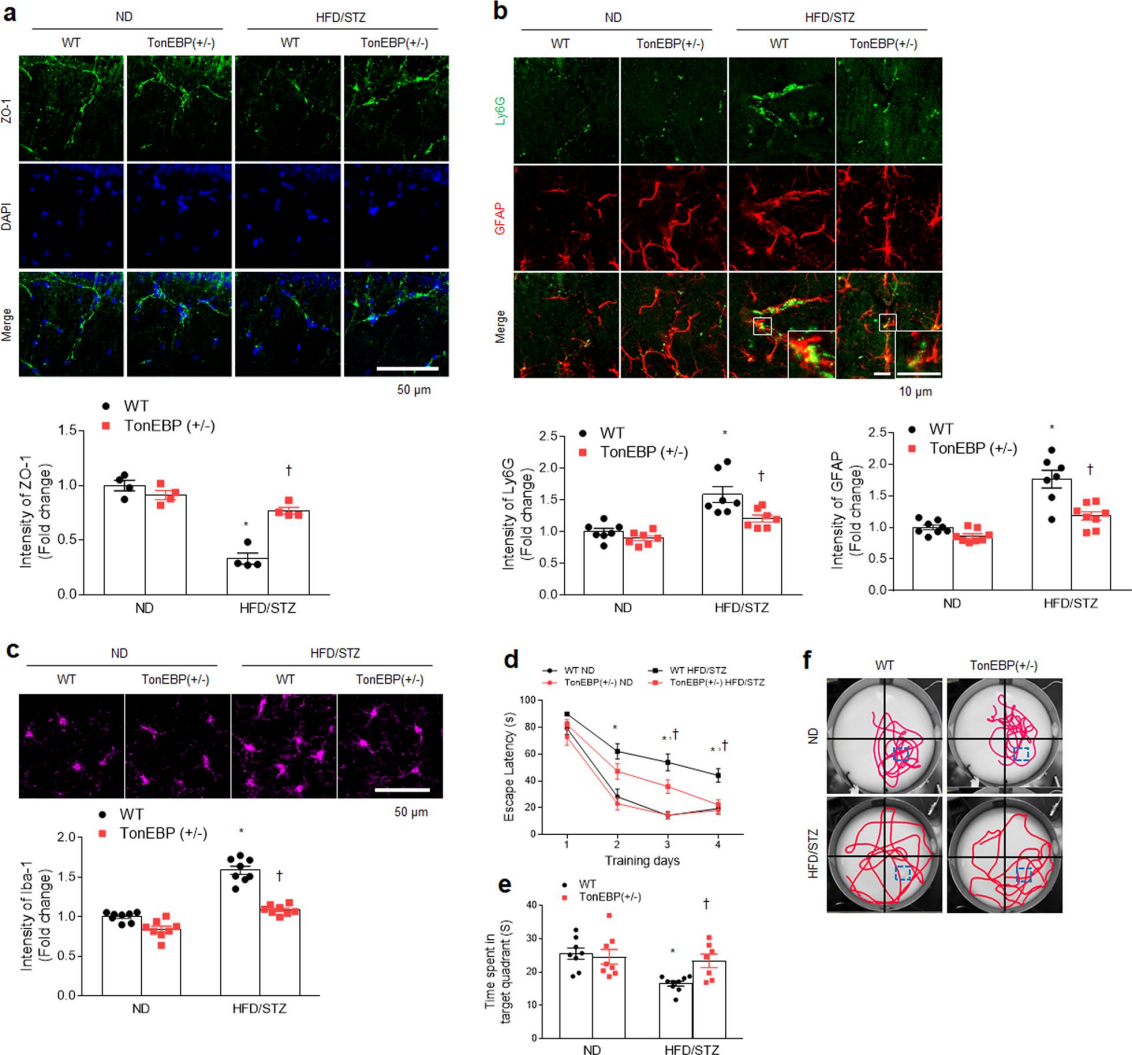
TonEBP haploinsufficiency attenuates BBB leakage and neuroinflammation and enhances memory deficits in HFD/STZ-induced diabetic mice.** a** Representative immunofluorescence staining for ZO-1 in the hippocampus CA1 region sections. DAPI was used for nuclear staining. Quantification of ZO-1-immunostained density in the images (*n* = 3–4, *F* = 35.86, *p* < 0.001). **b** Representative double immunofluorescence staining for Ly6G and GFAP in the hippocampus CA1 region sections. Quantification of Ly6G (*F* = 3.215, *p* = 0.086)—or GFAP (*F* = 8.746, *p* = 0.006)—immunostained density in the images (*n* = 3–4). **c** Representative immunofluorescence staining for Iba-1 in the hippocampus CA1 region sections. Quantification of Iba-1-immunostained density in the images (*n* = 3–4, *F* = 22.37, *p* < 0.001). **d** Escape latency over four days of Morris water-maze training (*n* = 7–9, *F* = 1.926, *p* = 0.047). **e** Time within the target platform quadrant (*n* = 7–9, *F* = 5.15, *p* = 0.031). **f** Representative swim paths during probe trials. Square boxes indicate the location of the hidden platform. Data are shown as mean ± SEM. The indicated *p* values represent a two-way ANOVA followed by Tukey’s post-hoc test. *p < 0.05 versus WT ND. ^†^*p* < 0.05 versus WT HFD/STZ

We examined whether TonEBP haploinsufficiency affected diabetic encephalopathy in the mouse hippocampus. TonEBP haploinsufficiency significantly reversed the reduced zonula occludens-1 (ZO-1) protein in the diabetic hippocampus (Fig. 4a). In addition, double immunofluorescent images showed that many Ly6G-positive neutrophils were detected in both intravascular and extravascular space of vessels ensheathed by GFAP-expressing perivascular astrocyte end-feet in HFD/STZ-induced diabetic hippocampus (Fig. 4b). Diabetic TonEBP (+ / −) mice had less Ly6G-positive neutrophils and glial activation (GFAP and Iba1) in the hippocampus compared to diabetic WT mice (Fig. 4b, c). As expected, the water–-maze test showed memory deficits in diabetic WT mice were improved by TonEBP haploinsufficiency (Fig. 4d–f). Taken together, these findings indicate that TonEBP haploinsufficiency could protect against diabetic encephalopathy including BBB impairment, neuroinflammation, and memory deficits.

### TonEBP haploinsufficiency attenuates hippocampal LCN2 and HO-1 expression in diabetic mice

**Fig. 5:**
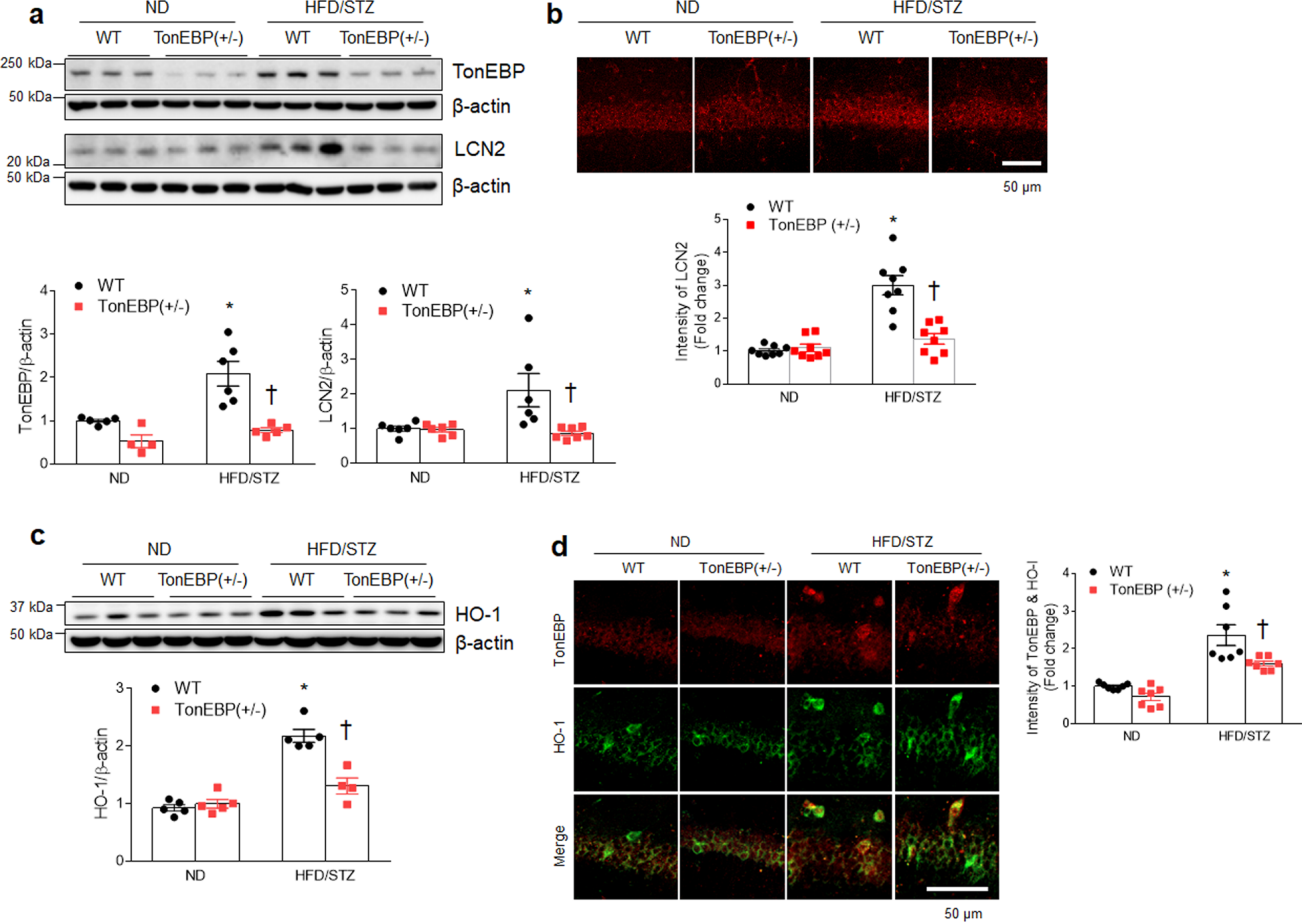
TonEBP haploinsufficiency attenuates hippocampal LCN2 and HO-1 expression in HFD/STZ-induced diabetic mice.** a** Western blots and quantified hippocampal expression of TonEBP (*F* = 4.977, *p* = 0.04) and LCN2 (*F* = 6.447, *p* = 0.019). Band intensities were normalized to β-actin (*n* = 4–7). **b** Representative immunofluorescence staining for LCN2 in the hippocampal CA1 region sections. Quantification of LCN2-immunostained density in the images (*n* = 3–4, *F* = 22.83, *p* < 0.001). **c** Western blots and quantified hippocampal expression of HO-1 (*n* = 4–5, *F* = 23.54, *p* < 0.001). Band intensities were normalized to β-actin. **d** Representative double immunofluorescence staining for TonEBP and HO-1 in hippocampal CA1 region sections. Quantification of co-localized TonEBP and HO-1-immunostained density in the images (*n* = 3–4, *F* = 2.525, *p* = 0.125). Data are shown as mean ± SEM. The indicated *p* values represent a two-way ANOVA followed by Tukey’s post-hoc test. *p < 0.05 versus WT ND. ^†^*p* < 0.05 versus WT HFD/STZ

Consistent with the reduced LCN2 levels in serum and adipose tissues in diabetic TonEBP (+ / −) mice, the increased hippocampal LCN2 level in diabetic WT mice were also reduced by TonEBP haploinsufficiency (Fig. 5a, b). Notably, we found that nuclear TonEBP and cytosolic LCN2-positive cells were observed in the same neurons and these neurons were markedly observed in hippocampal CA1 region of HFD/STZ-treated WT mice (Additional file 1: Fig. S6). However, TonEBP-positive cells were not co-localized with GFAP-positive astrocytes in the diabetic hippocampus. TonEBP haploinsufficiency attenuated increased hippocampal HO-1 protein in diabetic WT mice (Fig. 5c). Furthermore, the increased number of HO-1-positive cells, which are colocalized with TonEBP-positive neurons in diabetic WT mice, was prominently reduced by TonEBP haploinsufficiency (Fig. 5d).

### Elevated LCN2 and TonEBP levels are associated with upregulation of NF-κBp65 in LPS/HG-treated RAW 264.7 macrophages

**Fig. 6:**
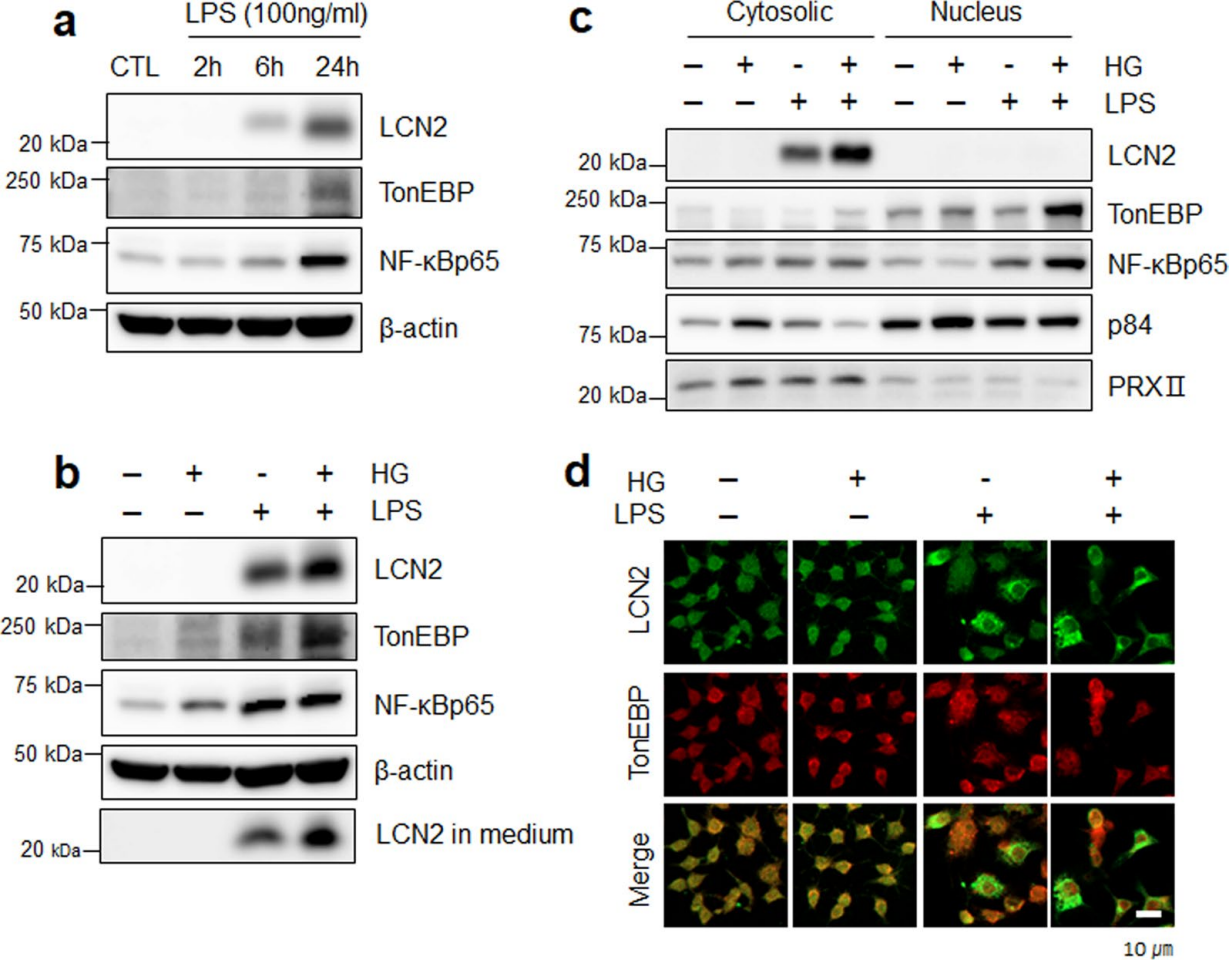
Expression of LCN2 and TonEBP in LPS-treated RAW 264.7 macrophages is enhanced by HG treatment.** a** Western blot analysis of LCN2, TonEBP, and NF-κBp65 at 2, 6, 24 h after LPS treatment. **b** Western blot analysis of LCN2, TonEBP, NF-κBp6, and supernatant medium LCN2 in LPS-treated RAW 264.7 macrophages following HG treatment. **c** Western blot analysis of LCN2, TonEBP, and NF-κBp65 in cytosolic and nuclear fractions of LPS or LPS/HG-treated RAW 264.7 macrophages. p84 and PRXII are nuclear and cytosolic markers, respectively. **d** Representative immunofluorescence staining for LCN2 and TonEBP in CTL, HG, LPS, and LPS/HG-treated RAW 264.7 macrophages

The expression of proinflammatory genes by LPS treatment in RAW 264.7 cells has previously been reported to be induced by TonEBP and NF-κBp65 [32, 33]. We first treated RAW 264.7 macrophages with LPS and HG to induce inflammation and hyperglycemia. LCN2, TonEBP, and NF-κBp65 proteins gradually increased after LPS treatment in a time-dependent manner (Fig. 6a). Notably, LPS-induced LCN2 and TonEBP expressions were promoted by HG treatment in RAW 264.7 cells, and LCN2 levels in media were also highly increased (Fig. 6b)**.** As expected, cytosolic LCN2, nuclear TonEBP, and NF-κBp65 significantly increased in response to LPS and were stimulated by HG treatment (Fig. 6c, d). These data indicate that the upregulation of TonEBP under HG-induced hyperglycemic conditions could promote LCN2 expression through NF-κBp65-mediated signaling.

### TonEBP promotes the production of LCN2 protein in HG-treated mouse hippocampus HT22 cells

**Fig. 7:**
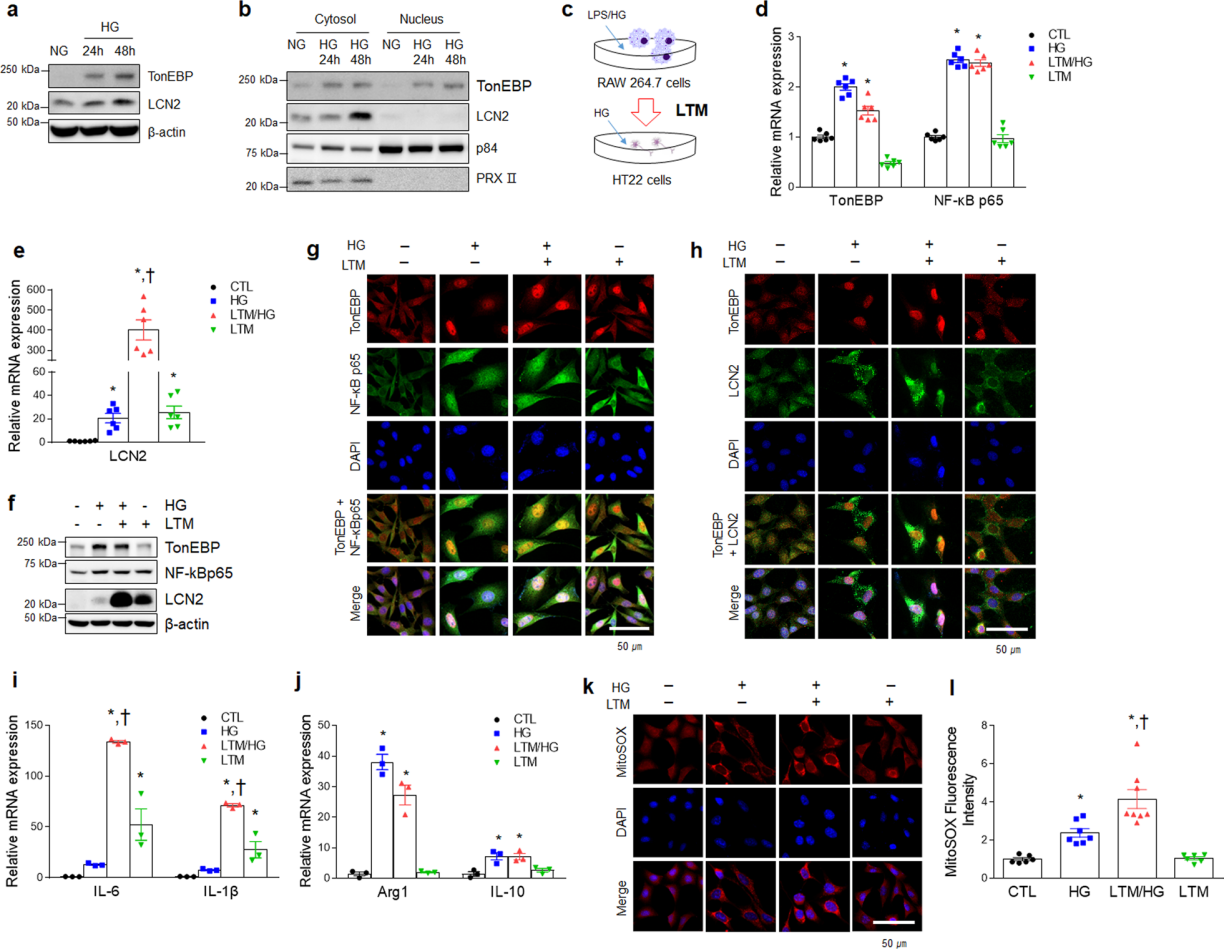
Expression of TonEBP and LCN2 in HG-treated HT22 cells is increased by LPS/HG-treated RAW 264.7 macrophage medium (LTM).** a** Western blot analysis of TonEBP and LCN2 in HT22 cells after HG treatment. **b** Western blot analysis of TonEBP and LCN2 in the cytosolic and nuclear fractions of HG-treated HT22 cells. p84 and PRXII are nuclear and cytosolic markers, respectively. **c** A schematic drawing of the LPS/HG-treated RAW 264.7 macrophage medium **(**LTM) treatment method in HG pre-treated HT22 cells. **d**, **e** Expression of mRNA encoding TonEBP (*F* = 124.9, *p* < 0.001) and NF-κBp65 (*F* = 204.9, *p* < 0.001) (d), LCN2 **e** (*F* = 59.14, *p* < 0.001) in HG, LTM/HG-treated HT22 cells was measured by quantitative RT-PCR (*n* = 6). **f** Western blot analysis of TonEBP, NF-κBp65, and LCN2 in LTM/HG-treated HT22 cell. **g**, **h** Representative immunofluorescence staining for NF-κBp65 and TonEBP (g) and LCN2 and TonEBP (h) in LTM/HG-treated HT22. DAPI was used for nuclear staining. **i**, **j** Expression of mRNA encoding IL-6 (*F* = 59.83, *p* < 0.001) and IL-1β (F = 58.6, *p* < 0.001) (**i**), Arg1 (*F* = 79.87, *p* < 0.001) and IL-10 (*F* = 10.29, *p* = 0.004) **j** in LTM/HG-treated HT22 cells was measured by quantitative RT-PCR (*n* = 3). **k** Detection of superoxide by MitoSOX™ Red in LTM/HG-treated HT22 cells. DAPI was used for nuclear staining. **l** Quantification of MitoSOX™ Red fluorescence intensity (*F* = 21.92, *p* < 0.001). Data are shown as mean ± SEM. The indicated *p* values represent a one-way ANOVA followed by Tukey’s post-hoc test. *p < 0.05 versus CTL, ^†^*p* < 0.05 versus HG-treated HT22 cells

To verify whether circulating inflammatory mediators stimulate neuroinflammatory responses, we next tested the effects of inflammation on HG-pretreated HT22 cells together with medium (LTM) obtained from LPS/HG-treated RAW 264.7 cells. Forty-eight hours after HG treatment, the expressions of TonEBP and LCN2 were markedly increased in HT22 cells (Fig. 7a). Consistent with the findings in RAW 264.7 cells, there was also a significant increase in nuclear TonEBP and cytosolic LCN2 expression in HG-treated HT22 cells (Fig. 7b). To determine whether LCN2 secreted from LPS/HG-treated RAW 264.7 cells affected the expression of TonEBP, LCN2, and NF-κBp65 in HG-treated HT22 cells, we administered LTM to HG-treated HT22 cells (Fig. 7c). Although TonEBP and NF-κBp65 mRNA levels in these HG-treated HT22 cells were not affected by LTM treatment, LCN2 mRNA was significantly increased in LTM/HG-treated HT22 cells compared to only LTM or HG-treated HT22 cells (Fig. 7d–f). In addition, nuclear TonEBP and NF-κBp65 were co-localized in LTM/HG-treated HT22 cells compared to either HG- or LTM-treated HT22 cells, and cytosolic LCN2 was markedly elevated in LTM/HG-treated HT22 cells (Fig. 7g. h). Furthermore, *IL-6* and *IL-1β* mRNA were increased in LTM/HG-treated HT22 cells compared to HG-only or LTM-only treatments, and the mRNA levels of *Arg1* and *IL-10* were decreased in LTM/HG-treated HT22 cells (Fig. 7i, j). The staining intensities of mitochondrial superoxide (MitoSOX) were the highest in LTM/HG-treated HT22 cells (Fig. 7k, l). These findings suggest that elevated TonEBP could promote the induction of LCN2-mediated inflammation in mouse hippocampal cells.

### TonEBP inhibition attenuates increased LCN2 expression in LTM/HG-treated HT22 cells

**Fig. 8:**
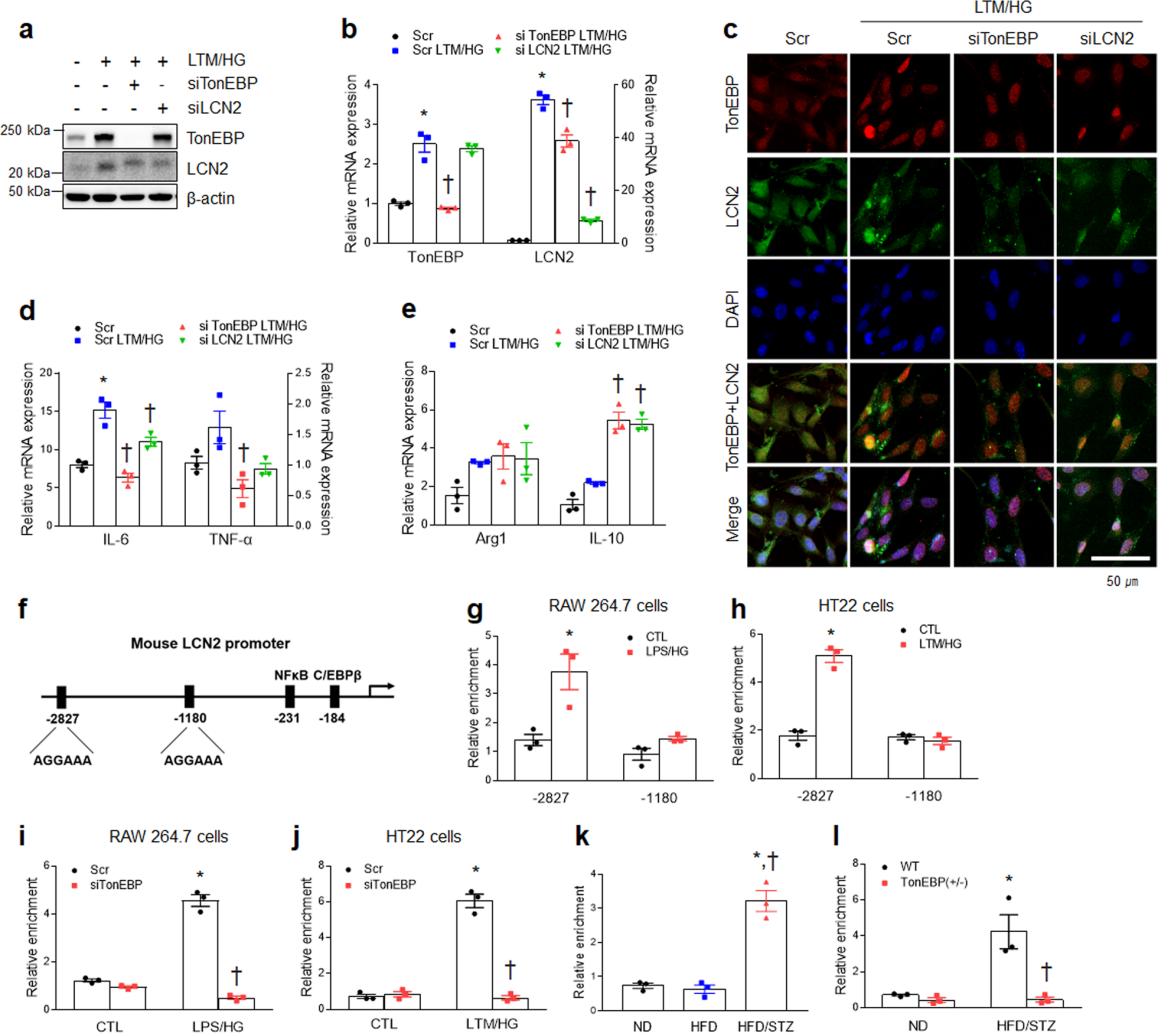
TonEBP modulates LCN2 gene expression under inflammatory and hyperglycemic LTM/HG-treated conditions in vitro and in vivo. **a** Western-blot analysis of TonEBP and LCN2 in HT22 cells transfected with Scramble (Scr) or TonEBP or LCN2 siRNAs. **b** Expression of mRNA encoding TonEBP (*F* = 59.76, *p* < 0.001) and LCN2 (*F* = 290.1, *p* < 0.001) in LTM/HG-treated HT22 cells was measured by quantitative RT-PCR. **c** Representative immunofluorescence staining for TonEBP and LCN2 in LTM/HG-treated HT22 cells transfected with Scr or TonEBP or LCN2 siRNA. DAPI was used for nuclear staining. **d**, **e** Expression of mRNA encoding IL-6 (*F* = 84.22, *p* < 0.001) and TNF-α (*F* = 6.274, *p* = 0.017) (**d**), or Arg1 (*F* = 2.783, *p* = 0.11) and IL-10 (*F* = 56.45, *p* < 0.001) **e** in LTM/HG-treated HT22 cells was measured by quantitative RT-PCR. **f** A schematic drawing of the TonEBP binding motif in the mouse *LCN2* promoter. **g**, **h** ChIP assay for TonEBP in the *LCN2* promoter region as indicated in LPS/HG-treated RAW 264.7 macrophages **g** (F = 7.123, *p* = 0.028) or LTM/HG-treated HT22 **h** (*F* = 85.04, *p* < 0.001) cells**. i**, **j** ChIP assay for TonEBP at the − 2827 location in the *LCN2* promoter in LPS/HG-treated RAW 264.7 macrophage **i** (*F* = 202.2, *p* < 0.001) or LTM/HG-treated HT22 **j** (*F* = 161.4, *p* < 0.001) cells transfected with TonEBP siRNA. **k**–**l** ChIP assay for TonEBP at the − 2827 location in the *LCN2* promoter in WT mice **k** (*F* = 55.79, *p* < 0.001) or TonEBP ( ±) **l** (*F* = 12.94, *p* = 0.007). Data (*n* = *3*) are shown as mean ± SEM. The indicated *p *values represent a one-way ANOVA in **c**, **e**, **f**, and **l,** or a two-way ANOVA in **h**, **i**, **j**, **k**, and **m** followed by Tukey’s post-hoc test. *p < 0.05 versus Scr or CTL or WT ND, ^†^*p* < 0.05 versus LTM/HG-treated HT22 cells or WT HFD/STZ

We next investigated the effects of TonEBP knockdown on LCN2-mediated inflammation in LTM/HG-treated HT22 cells using a siRNA approach. As expected, TonEBP knockdown reduced LCN2 protein and mRNA levels in LTM/HG-treated HT22 cells, but the TonEBP expression level was not affected by LCN2 depletion (Fig. 8a, b). In addition, the lack of TonEBP caused a decrease of cytosolic LCN2 proteins and nuclear TonEBP proteins in LTM/HG-treated HT22 cells (Fig. 8c). Furthermore, the proinflammatory mRNAs of *IL-6* and *TNF-α* in LTM/HG-treated HT22 cells were significantly decreased by TonEBP siRNA, while the anti-inflammatory mRNA for *Arg1* was not changed either by TonEBP or LCN2 siRNAs (Fig. 8d, e). However, TonEBP or LCN2 siRNAs enhanced increased *IL-10* mRNA in LTM/HG-treated HT22 cells.

To further investigate whether TonEBP transcriptionally regulated LCN2 expression via direct binding to its regulatory regions, we next tested whether TonEBP bound to the LCN2 promoter as a transcriptional factor using a ChIP analysis. Fujino et al. [34] reported NF-kBp65 binding to positions − 220 to − 231 of the LCN2 promoter (Additional file 1: Fig. S7a). As expected, we confirmed that NF-kB bound to the LCN2 promoter at positions − 220 to − 231, and that binding was increased in LPS/HG-treated RAW 264.7 cells and LTM/HG-treated HT22 cells (Additional file 1: Fig. S7b, c). We also found that TonEBP is specifically bound to a consensus sequence (positions − 2827 and − 1180) in the mouse LCN2 promoter (Fig. 8f). Indeed, TonEBP binding to the − 2827 position of the LCN2 promoter region was significantly increased in LPS/HG-treated RAW 264.7 cells and LTM/HG-treated HT22 cells, respectively (Fig. 8g, h). Furthermore, TonEBP knockdown reduced this binding (Fig. 8i, j). In hippocampal tissues, we also found that TonEBP bound to the LCN2 promoter in HFD/STZ-induced diabetic mice, and this binding was abolished by TonEBP haploinsufficiency (Fig. 8k, l). Taken together, these results suggest that the TonEBP may transcriptionally regulate systemic and neuroinflammation via the direct binding to position − 2827 of the LCN2 promoter region under diabetic conditions of low-grade inflammation and hyperglycemia.

## Discussion

**Fig. 9:**
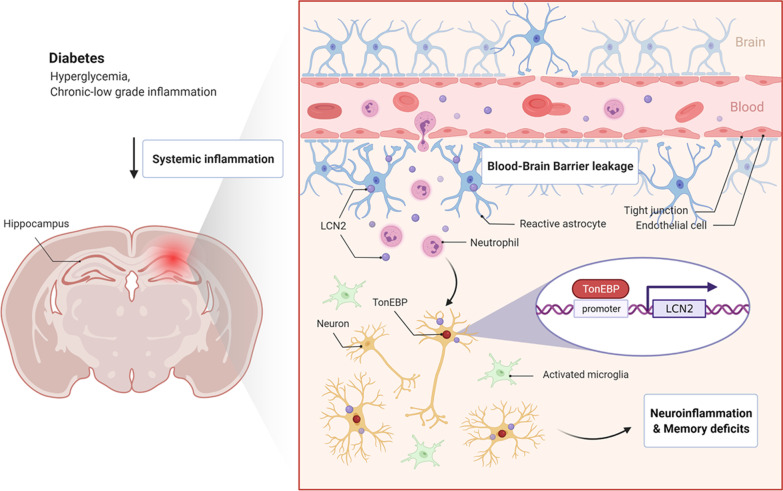
Graphical summary of the proposed role of TonEBP in diabetes-induced neuroinflammation and memory deficits. During chronic diabetic conditions, systemic inflammation causes infiltration of neutrophils through blood–brain barrier leakage. This LCN2 production during chronic diabetes promotes TonEBP-mediated neuroinflammation, ultimately leading to memory deficits. In particular, TonEBP transcriptionally regulates LCN2 protein levels under diabetic conditions

We have demonstrated three major findings: (i) circulating levels of LCN2 and TonEBP, as pro-inflammatory mediators, increase in diabetic mice or patients with memory deficits; (ii) TonEBP haploinsufficiency attenuates macrophage infiltrations, BBB breakdown, neuroinflammation, and memory deficits in HFD/STZ-induced diabetic mice; and (iii) TonEBP transcriptionally regulates LCN2 protein levels under diabetic conditions. Based on these results, the functional regulation of LCN2 as a proinflammatory factor in the progression of diabetic encephalopathy could be regulated by TonEBP (Fig. 9).

Patients with T2DM have been reported to have experienced a state of obesity associated with insulin resistance, and hyperinsulinemia as a result of activated β-cell compensation [35]. However, the development of T2DM ultimately leads to a failure of pancreatic β-cells in its late stages, and these physiological and pathological conditions usually appear in older patients with T2DM. Notably, relative insulin deficiency is closely associated with cognitive impairment in AD patients [36]. Several studies have indicated that neutrophils reacting to inflammatory stimuli (ionomycin and LPS) generate oxidative stress in addition to releasing inflammatory cytokines triggered by high glucose in T2DM [37, 38]. We, therefore, designed experiments to mimic these pathological states (chronic low-grade inflammation and hyperglycemia) of T2DM with a reduction in functional β-cell mass. In addition, we selected male mice to reduce variations in their experimental results without influence of the estrogen cycle and adiposity of female animals [39, 40]. Our findings showed that, compared to HFD-only fed mice, HFD/STZ-induced diabetic mice had typical diabetic complications including necrotic adipocytes with neutrophil and macrophage infiltrations, hepatic steatosis, insulin resistance, neuroinflammation, as well as memory deficits. Notably, we found that HFD/STZ-induced diabetic mice had more serum, adipose tissue, and hippocampal levels of LCN2 and TonEBP relative to HFD-fed mice. Taken together, these findings indicate that under chronic diabetic conditions, both LCN2 and TonEBP may play crucial roles as mediators of diabetic encephalopathy.

Under chronic diabetic conditions, an inflammatory response usually involves an accumulation of neutrophils and macrophages that breaks down necrotic adipose tissues and stimulates a curative response [41, 42]. In particular, neutrophils are likely involved in stimulating macrophage activity through the production of soluble LCN2 that is required for efficient non-alcoholic steatohepatitis healing [43]. Neutrophils expose to high glucose concentrations in HFD-fed mice, and neutrophil-derived elastase promotes the activation and infiltration of M1-like macrophages into adipose tissue [44–46], and NE-deficient obese mice have been shown to exhibit reduced numbers of macrophages and reduced inflammatory cytokines in adipose tissue [47]. In this study, we have demonstrated that the marked increases in TonEBP- and LCN2-positive inflammatory cells (F4/80 and CD68 for macrophages; Ly6G and NE for neutrophils) in free perilipin 1-positive necrotic adipocytes from diabetic WT mice were reversed by TonEBP haploinsufficiency. In addition, we have shown that co-treatment with LPS and HG promoted increased expressions of TonEBP, LCN2, and NF-κBp65 in RAW 264.7 macrophages. In support of these present findings, HFD-fed obese LCN2 (− / −) mice or HFD/STZ-treated diabetic TonEBP (+ /−) mice had decreased TNF-α and IL-6 levels in their adipose tissues [20, 24]. These findings are consistent with the evidence that hyperglycemia-induced TonEBP caused the upregulation of NF-κB activity and M1-like macrophage polarization [23]. Myeloid cell-specific deletion of TonEBP has also been shown to result in milder inflammation and sepsis in LPS-treated mice [32]. Kwon and colleagues have reported that TonEBP promoted inflammation by inhibiting IL-10, inhibiting M2 expression, and conversely, increasing M1 gene expression and inhibiting the transcriptional regulation of Nrf2, thereby inhibiting HO-1 expression [32, 48, 49]. Our findings are consistent with the evidence that diabetes-induced TonEBP caused the upregulation of NF-κB activity and M1 polarization [23]. On the other hand, although HO-1 has been implicated in the cytoprotective defense response against various oxidative and inflammatory insults, sustained induction of HO-1 can be detrimental and lead to a low-grade inflammatory state in diabetes [50]. Our result indicates that chronic low-grade inflammation causes sustained HO-1 production in the diabetic hippocampus as well as TonEBP upregulation. Taken together, the data indicate that the elevation of TonEBP levels under diabetic conditions promoted M1-like macrophages and local inflammation, further supporting the role of TonEBP in chronic diabetic conditions, and exacerbated systemic inflammation via the upregulation of LCN2 through an NF-κBp65-dependent mechanism.

It has been widely reported that elevated levels of inflammatory mediators in patients with diabetes promote neuroinflammation by triggering detrimental neutrophil/microglia activations in the diabetic brain [3, 51]. Our previous studies showed that resveratrol or caloric restriction improved not only insulin sensitivity and peripheral inflammation but also neuroinflammation and memory deficits in diabetic mice [7, 8]. Furthermore, we have demonstrated that caloric restriction attenuated the higher LCN2 levels in the serum and the hippocampus of ob/ob mice, and led to improvements in their memory deficits [12]. The upregulation of LCN2 in the brain can be induced by peripheral turpentine-induced inflammation [38], and increased circulating levels of LCN2 have been closely associated with MCI in AD [52]. As expected, we also found increased levels of LCN2 in serum samples from diabetic human subjects with low K-MMSE scores. Despite TonEBP not being secreted and released as an LCN2 adipokine, to our knowledge, we are the first to report TonEBP serum levels for both the mouse and humans using highly sensitive ELISAs. We maybe suggest that high levels of circulating TonEBP are linked to the release from necrotic adipocytes under chronic diabetic conditions. Therefore, we strongly suggest that both LCN2 and TonEBP are systemic inflammatory mediators that play a causative role in diabetic encephalopathy. Specifically, TonEBP haploinsufficiency attenuated Ly6G-positive neutrophils through BBB leakage in the hippocampal CA1 region of diabetic WT mice (Fig. 4a, b). Consistent with the in vivo results, in vitro analyses also showed that in HG-pretreated mouse hippocampal HT22 cells, LTM containing LCN2 from LPS/HG-treated RAW 264.7 cells increased proinflammatory cytokines (IL-6 and IL-1β), NF-κBp65, and MitoSOX levels. Taken together, these findings indicate that LCN2 may exert a positive TonEBP-mediated NF-κBp65 signal at high blood–glucose concentrations. We, therefore, strongly suggest that LCN2 could be the systemic inflammatory mediator that plays a causative role in neuroinflammation in the diabetic brain via BBB breakdown.

Our previous studies have reported that upregulating systemic and hippocampal LCN2 levels caused hippocampal atrophy and obesity-induced cognitive deficits in ob/ob mice [12, 13]. While LCN2 deficiency has been shown to improve insulin resistance and diabetic encephalopathy [20, 53], others have found that LCN2 (-/-) mice were more susceptible to HFD-induced obesity and LPS-induced sepsis [19, 22], and one study has suggested that LCN2 deficiency plays only a minor role in mice fed with HFD [54]. Thus, these paradoxical roles of LCN2 in metabolic and inflammatory disorders should be documented to fully understand diabetic complications. In the present study, we further determined the functional role of LCN2 and its pathways in the progression of neuroinflammation and memory deficits in the diabetic brain. Most studies have shown that LCN2 is expressed in capillaries, microglia, and astrocytes, but rarely in neurons [4, 14, 55, 56]. However, other investigators have shown that neuronal LCN2 expression increased after stroke in humans, in a rat cerebral–ischemia model, and in ob/ob mice [13, 57, 58]. The neuronal LCN2 labeling in the diabetic hippocampus and in HG-pretreated HT22 cells decreased in TonEBP (+ / -) mice and after TonEBP siRNA treatment, respectively. Based on these findings, we suggest that as an inflammatory mediator, LCN2 crosses the BBB breakdown and activates the TonEBP- or LCN2-positive neurons as paracrine or autocrine in the diabetic brain. Our results showed that TonEBP siRNA inhibited LCN2 expressions in LTM/HG-treated HT22 cells. Previous studies have supported that neuronal TonEBP is upregulated following systemic hypertonicity and hyperglycemia [24, 25]. Thus, these findings for restricted localization of LCN2 and TonEBP to neurons raise questions about their binding in neurons. Interestingly, a putative TonEBP DNA binding sequence has been reported in a region of the human LCN2 gene [59, 60]. Our ChIP assay showed that TonEBP binding increased at position –2827 of the LCN2 promoter region both in vitro and in vivo. Taken together, we suggest, for the first time, that under diabetic conditions, elevated LCN2 and TonEBP in inflammatory cells adversely influence diabetic encephalopathy through BBB leakage.

## Conclusion

We propose a model for local adipose tissue inflammation and neuroinflammation through the interplay of both TonEBP and LCN2 in HFD/STZ-induced diabetic mice. Notably, we have shown for the first time that circulating levels of TonEBP and LCN2 are closely associated with memory deficits in diabetic mice and human patients. LCN2-positive neutrophils cross the BBB leakage and then this neuroinflammatory response is aggravated by TonEBP in diabetic hippocampal neurons. Therefore, these findings suggest that LCN2, as a proinflammatory mediator, exerts a local effect on systemic and neuroinflammation via the regulation of TonEBP-mediated NF-κBp65 signaling under chronic diabetic conditions.

## Supplementary Information

**Additional file 1: Fig. S1.** Increased weight and HAFLD in HFD-fed mice with or without STZ. **Fig. S2.** Insulin resistance in HFD-fed mice with or without STZ. **Fig. S3.** TonEBP and LCN2 expression in the hippocampus of HFD-fed mice with or without STZ. **Fig. S4.** TonEBP (+ / −) mice show ameliorated HFD/STZ-induced hepatic steatosis and eWAT inflammation. **Fig. S5.** TonEBP-and LCN2-positive neutrophils are observed in epididymal white adipose tissue of HFD/STZ-induced diabetic mice. **Fig. S6.** TonEBP-and LCN2-positive neurons are observed in hippocampal CA1 region of HFD/STZ-induced diabetic mice. **Fig. S7.** The TonEBP binding site in the mouse *LCN2* promoter. **Table S1.** List of primary antibodies. **Table S2.** List of qRT-PCR primers. **Table S3.** List of qRT-PCR primer for ChIP assay. **Table S4.** Clinical characteristics of normal subjects (CTL) and type 2 diabetic patients (DM) with or without mild cognitive impairment (MCI).

## Data Availability

The data that support the findings of this study are available from corresponding author on reasonable request.

## Abbreviations

BBB: Blood–brain barrier
DM: Type 2 diabetes mellitus
GFAP: Glial fibrillary acidic protein
HFD: High-fat diet
HO-1: Heme oxygenase-1
K-MMSE: Korean version of the Mini-Mental State Examination
LCN2: Lipocalin-2
MCI: Mild cognitive impairment
MitoSOX: Mitochondrial superoxide
NE: Neutrophil elastase
NF-κB: Nuclear factor-kB
RAGE: Receptor for advanced glycation end products
STZ: Streptozotocin
TonEBP: Tonicity-responsive enhancer-binding protein
VCAM1: Vascular cell adhesion molecule 1
ZO-1: Zonula occludens-1
