# Supplementary material for: Tonicity-responsive enhancer-binding protein promotes diabetic neuroinflammation and cognitive impairment via upregulation of lipocalin-2
Source: J Neuroinflammation. 2021 Nov 29;18:278. doi: 10.1186/s12974-021-02331-8 (PMC8628424; doi:10.1186/s12974-021-02331-8)
Supplement: Supplementary file 1 — Additional file 1: Fig. S1. Increased weight and HAFLD in HFD-fed mice with or without STZ. Fig. S2. Insulin resistance in HFD-fed mice with or without STZ. Fig. S3. TonEBP and LCN2 expression in the hippocampus of HFD-fed mice with or without STZ. Fig. S4. TonEBP (+ / −) mice show ameliorated HFD/STZ-induced hepatic steatosis and eWAT inflammation. Fig. S5. TonEBP-and LCN2-positive neutrophils are observed in epididymal white adipose tissue of HFD/STZ-induced diabetic mice. Fig. S6. TonEBP-and LCN2-positive neurons are observed in hippocampal CA1 region of HFD/STZ-induced diabetic mice. Fig. S7. The TonEBP binding site in the mouse LCN2 promoter. Table S1. List of primary antibodies. Table S2. List of qRT-PCR primers. Table S3. List of qRT-PCR primer for ChIP assay. Table S4. Clinical characteristics of normal subjects (CTL) and type 2 diabetic patients (DM) with or without mild cognitive impairment (MCI). [file 12974_2021_2331_MOESM1_ESM.docx]

**Tonicity-responsive enhancer-binding protein promotes diabetic neuroinflammation and cognitive impairment via upregulation of LCN2**

Eun Ae Jeong^1†^, Jaewoong Lee^1†^, Hyun Joo Shin^1^, Jong Youl Lee^1^, Kyung Eun Kim^1^, Hyeong Seok An^1^, Deok Ryong Kim^2^, Kyu Yeong Choi^3^, Kun Ho Lee^4,5^*, Gu Seob Roh^1^*

^1^Department of Anatomy and Convergence Medical Science, Bio Anti-aging Medical Research Center Institute of Health Sciences, College of Medicine, Gyeongsang National University, Jinju, 52727, Republic of Korea

^2^Department of Biochemistry, Institute of Health Sciences, College of Medicine, Gyeongsang National University, Jinju, 52727, Republic of Korea

^3^Gwangju Alzheimer’s Disease and Related Dementia Cohort Research Center, Chosun University, Gwangju 61452, Republic of Korea

^4^Department of Biomedical Science, Chosun University, Gwangju 61452, Republic of Korea

^5^Aging Neuroscience Research Group, Korea Brain Research Institute, Daegu 41062, Republic of Korea

†These authors contributed equally.

**
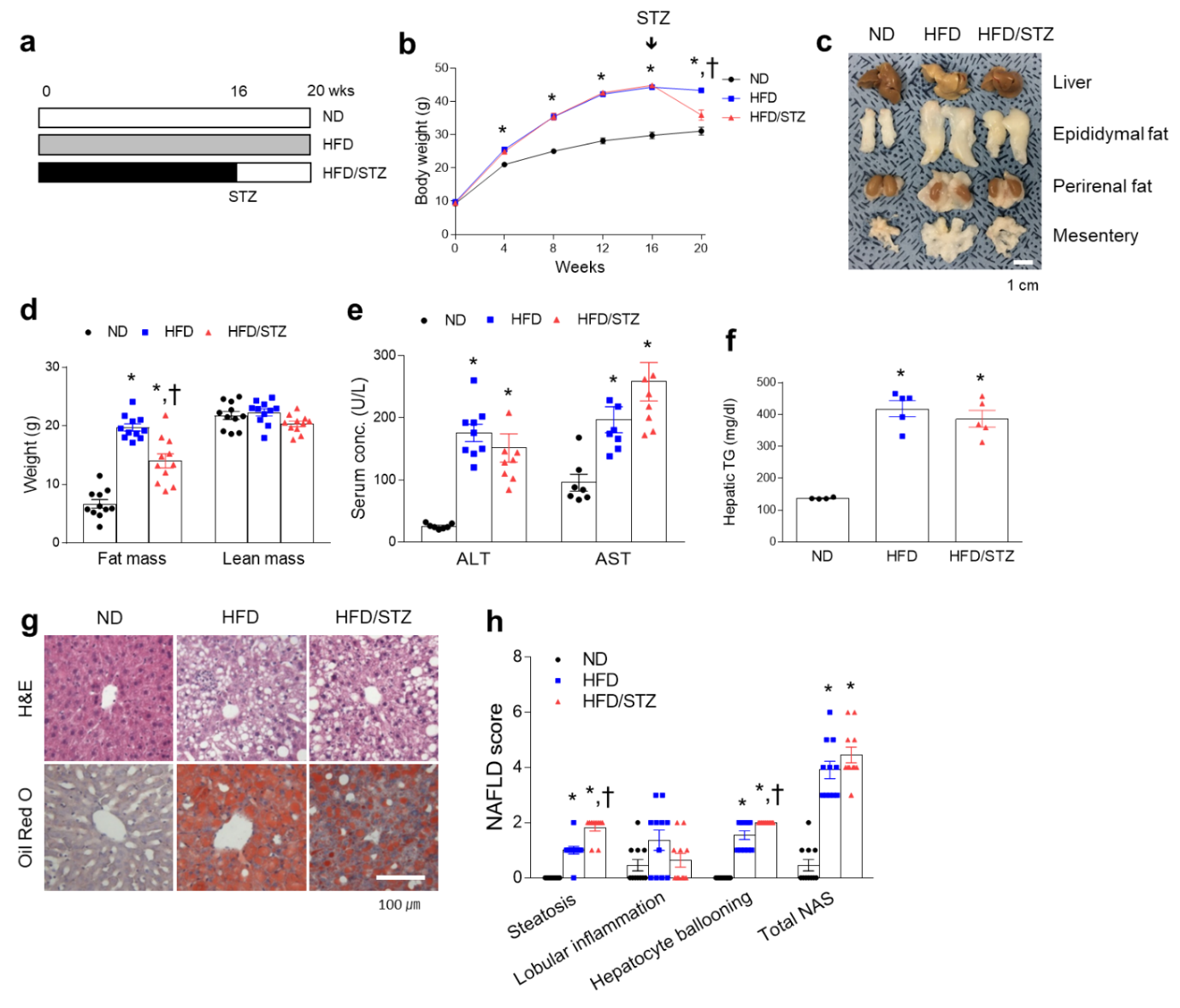
**

**Additional fie 1: Figure S1. Increased weight and HAFLD in HFD-fed mice with or without STZ. (a)** Experimental scheme for the diabetic-mouse models. Male mice were fed for 20 weeks with a high-fat diet (HFD; 60% Kcal fat) and were injected with streptozotocin (STZ; 100 mg/kg) at 16 weeks. Four weeks after the STZ injection, mice were sacrificed. **(b)** Body weights during the 20 weeks of diabetic development (*n* = 11, F = 21.45, *p* < 0.001). **(c)** Representative photographs of liver, epididymal fat pads, perirenal fat, and mesentery fat. **(d)** Fat (F = 55.32, *p* < 0.001) and lean (F = 2.984, *p* = 0.066) mass in normal diet (ND)- fed mice and HFD-fed mice with or without STZ (*n* = 11). **(e)** Serum hepatic enzymes (ALT; F = 21.09, *p* < 0.001 and AST; F = 10.84, *p* < 0.001) (*n* = 7-9). **(f)** Hepatic TG concentration levels (*n* = 4-5, F = 42.47, *p* < 0.001). **(g)** Representative images of H&E and Oil Red O staining) (*n* = 3-4). **(h)** Non-alcoholic fatty liver disease (NAFLD) activity scores from livers (Steatosis: F = 75.25, *p* < 0.001; Lobular inflammation: F = 2.958, *p* = 0.067; Hepatocyte ballooning: F = 133, *p* < 0.001; Total NAS: F = 63.73, *p* < 0.001). Data are shown as mean ± SEM. The indicated *p*-values represent a two-way ANOVA in b, or a one-way ANOVA in d, e, f, and h followed by Tukey’s post-hoc test. **p* < 0.05, versus ND. ^†^*p* < 0.05, versus HFD.


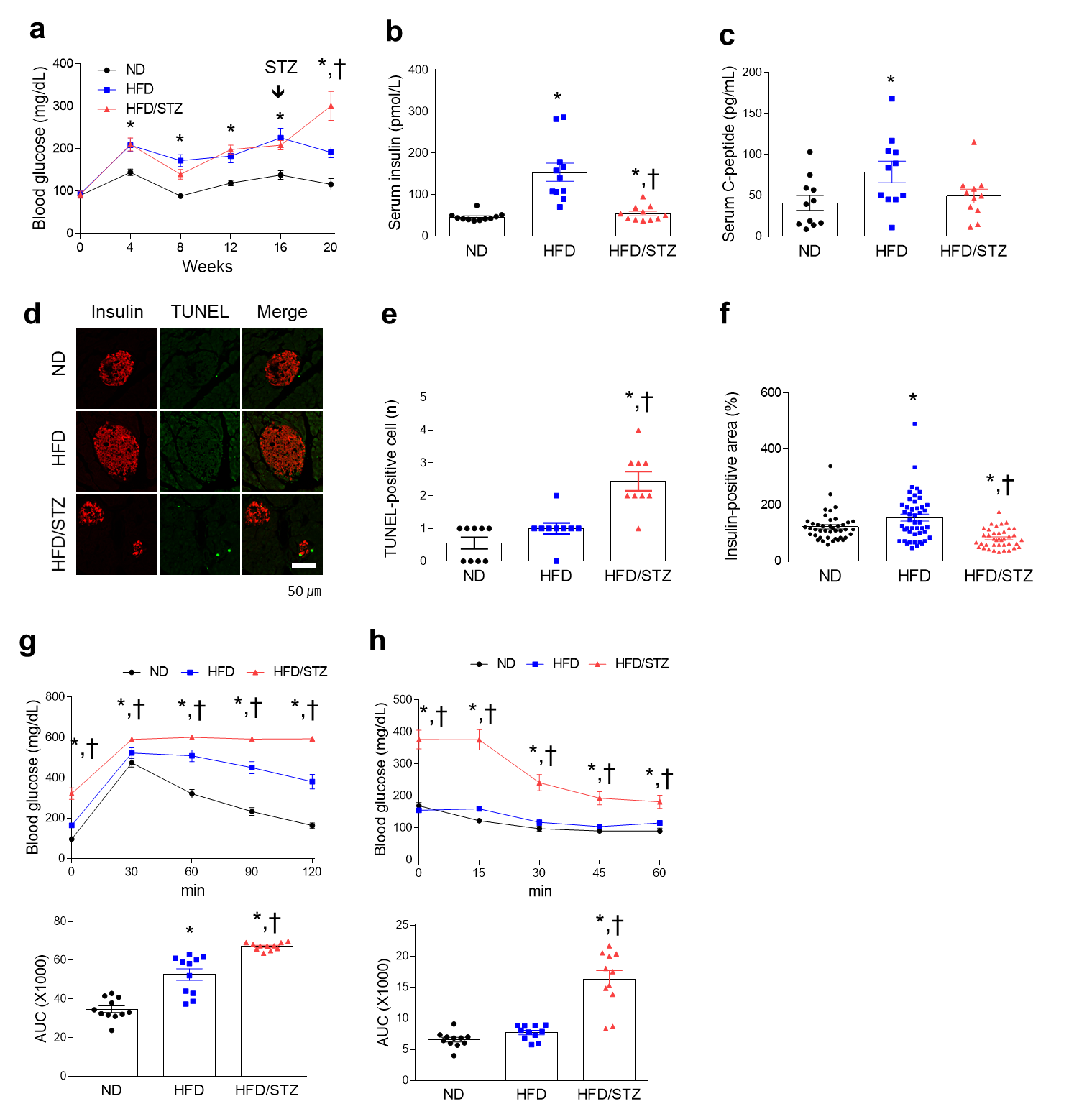


**Additional fie 1: Figure S2. Insulin resistance in HFD-fed mice with or without STZ.** **(a)** Fasting blood glucose levels during the 20 weeks of diabetic development (*n* = 11, F = 5.567, *p* < 0.001). **(b)** Serum insulin (*n* = 11, F = 20.95, *p* < 0.001). **(c)** Serum C-peptide (*n* = 11, F = 3.603, *p* = 0.04). **(d)** Representative images of immunostained insulin- and TUNEL-positive islet cells in pancreatic sections. The number of TUNEL **(e)** (F = 20.17, *p* < 0.001) - and insulin-positive cells **(f)** (F = 15.07, *p* < 0.001) in pancreatic sections (*n* = 3-4). **(g)** GTT (F = 9.796, *p* < 0.001) and area-under-the-curve (AUC) values (F = 65.09, *p* < 0.001) for the GTT (*n* = 11). (**h)** ITT (F = 6.476, *p* < 0.001) and AUC values (F = 38.74, *p* < 0.001) for the ITT (*n* = 11). Data are shown as mean ± SEM. The indicated *p*-values represent a repeated measure ANOVA (a, upper panel of g and h ), or a one-way ANOVA (b, c, e, f, lower panel of g and h) followed by Tukey’s post-hoc test. **p* < 0.05, versus ND. ^†^*p* < 0.05, versus HFD.

**
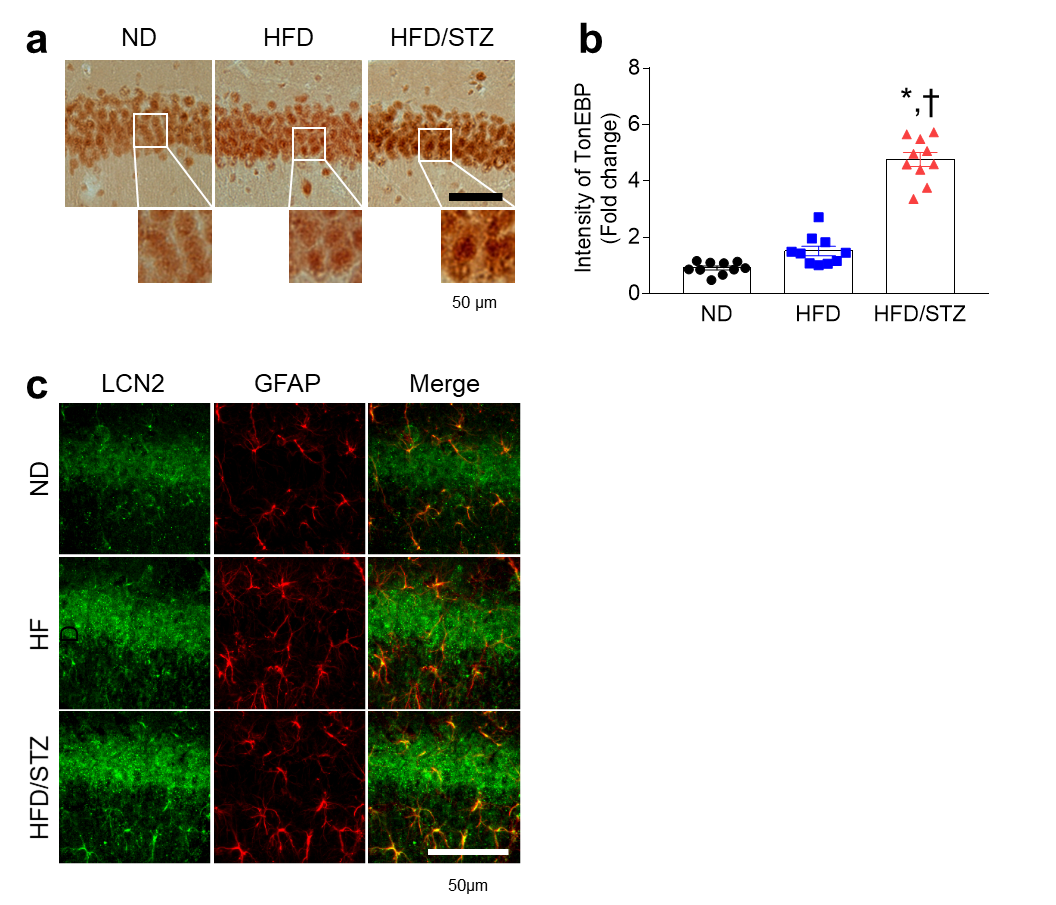
**

**Additional fie 1: Figure S3. TonEBP and LCN2 expression in the hippocampus of HFD-fed mice with or without STZ. (a)** Representative images of immunostained TonEBP-positive neurons in the hippocampus CA1 region section (*n* = 3-4). **(b)** Quantification of TonEBP-immunostained density in the images (F = 136.7, *p* < 0.001). **(c)** Representative immunofluorescence staining for LCN2 and GFAP in the hippocampal CA1 region sections. The indicated *p*-values represent a one-way ANOVA followed by Tukey’s post-hoc test. **p* < 0.05, versus ND. ^†^*p* < 0.05, versus HFD.

**
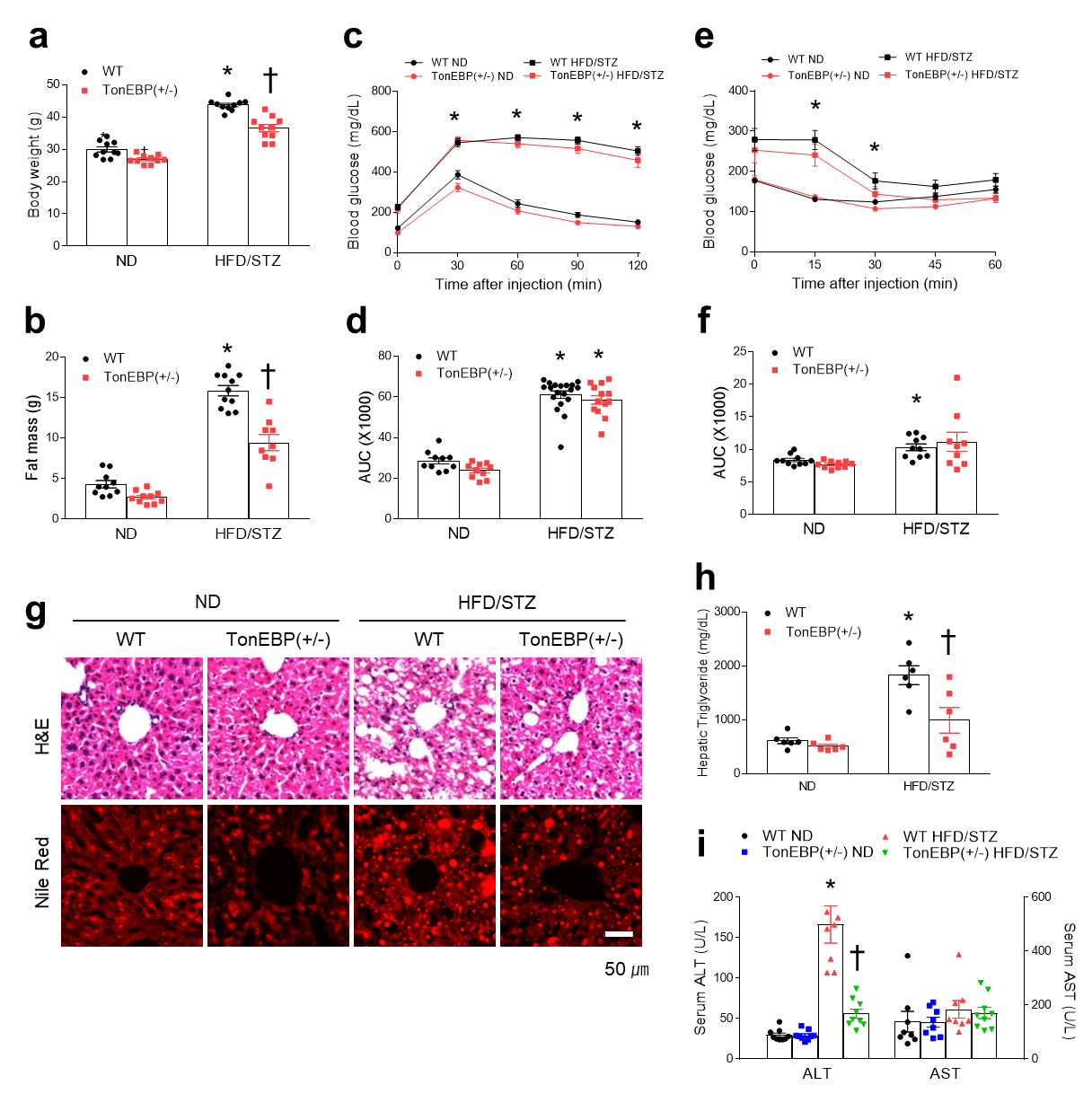
**

**Additional fie 1: Figure S4. TonEBP (+/**−**) mice show ameliorated HFD/STZ-induced hepatic steatosis and eWAT inflammation. (a** and **b)** Body weight (F = 7.15, *p* = 0.011) and fat mass (F = 5.392, *p* = 0.025) after 20 weeks of diabetic development (*n* = 10-19). **(c** and **d)** GTT (F = 11.49, *p* < 0.001) and AUC values (F = 0.3126, *p* = 0.579) for the GTT (*n* = 10-19). **(e** and **f)** ITT (F = 2.031, *p* = 0.22) and AUC values (F = 0.4667, *p* = 0.498) for the ITT (*n* = 10-19). **(g)** Representative H&E and Nile Red stained images (*n* = 3-4). **(h)** Hepatic TG concentration levels (*n* = 6, F = 5.982, *p* = 0.024). **(i)** Serum hepatic enzymes (ALT; F = 24.79, *p* < 0.001 and AST; F = 0.043, *p* = 0.837) (*n* = 8-9). Data are shown as mean ± SEM. The indicated *p*-values represent a two-way ANOVA followed by Tukey’s post-hoc test. **p* < 0.05, versus WT ND. ^†^*p* < 0.05, versus WT HFD/STZ.


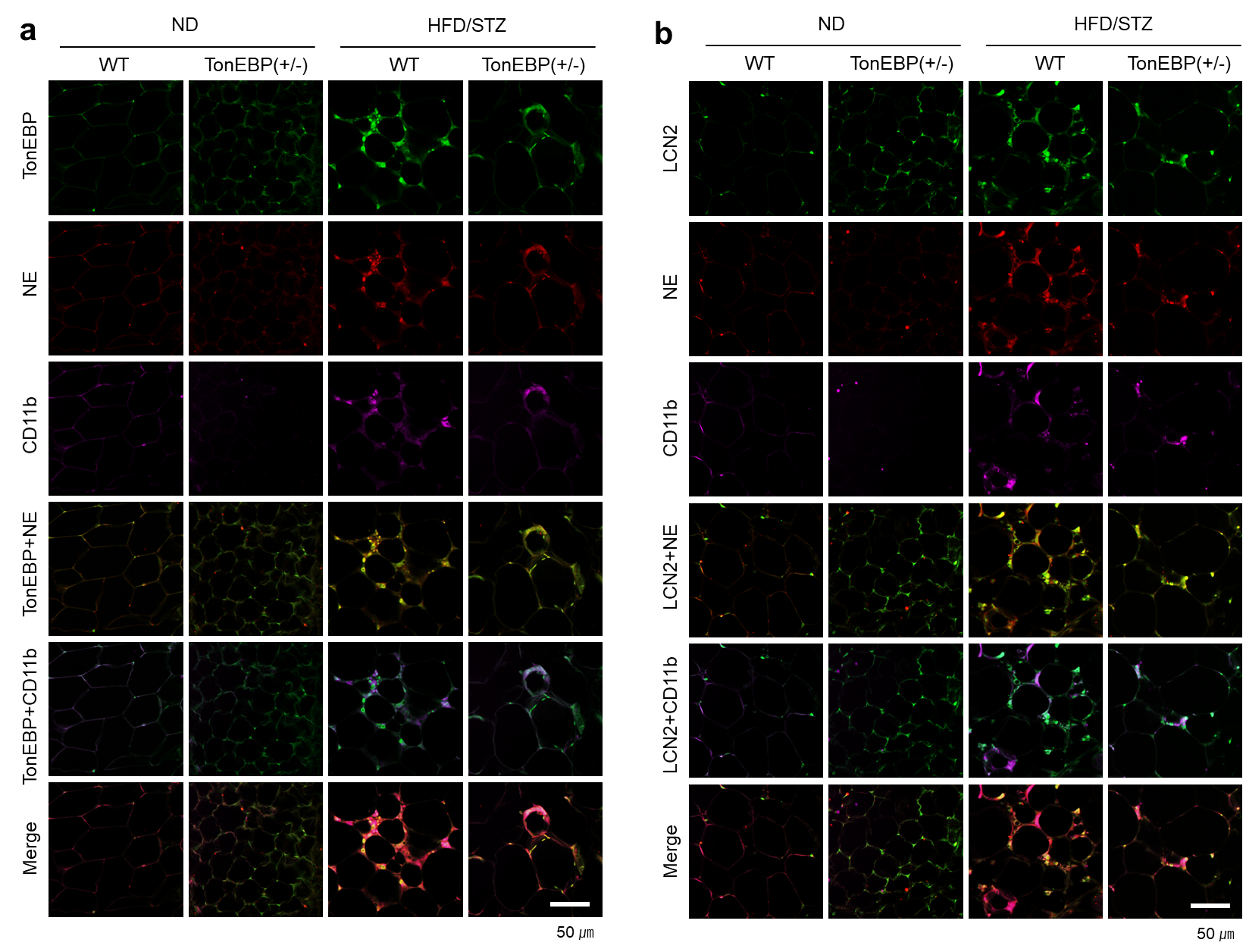


**Additional fie 1: Figure S5. TonEBP-and LCN2-positive neutrophils are observed in epididymal white adipose tissue of HFD/STZ-induced diabetic mice. (a)** Representative immunofluorescence staining for TonEBP, NE, and CD11b in adipose tissue sections. **(b)** Representative immunofluorescence staining for LCN2, NE, and CD11b in adipose tissue sections.


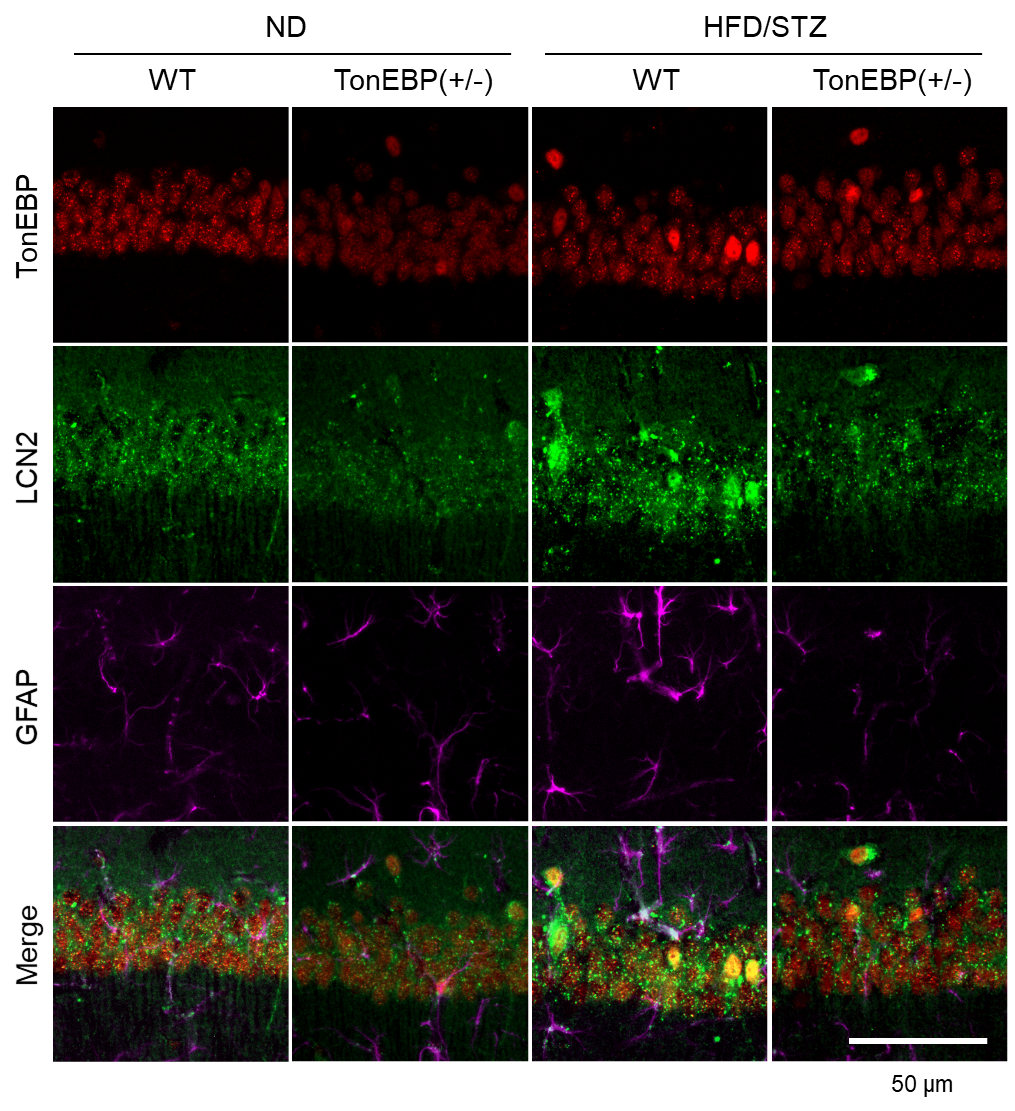


**Additional fie 1: Figure S6.** **TonEBP-and LCN2-positive neurons are observed in hippocampal CA1 region of HFD/STZ-induced diabetic mice.** Representative triple immunofluorescence staining for TonEBP, LCN2, and GFAP in hippocampal CA1 region sections. Both TonEBP and LCN2-positive cells are not co-localized with GFAP-positive astrocytes.

**
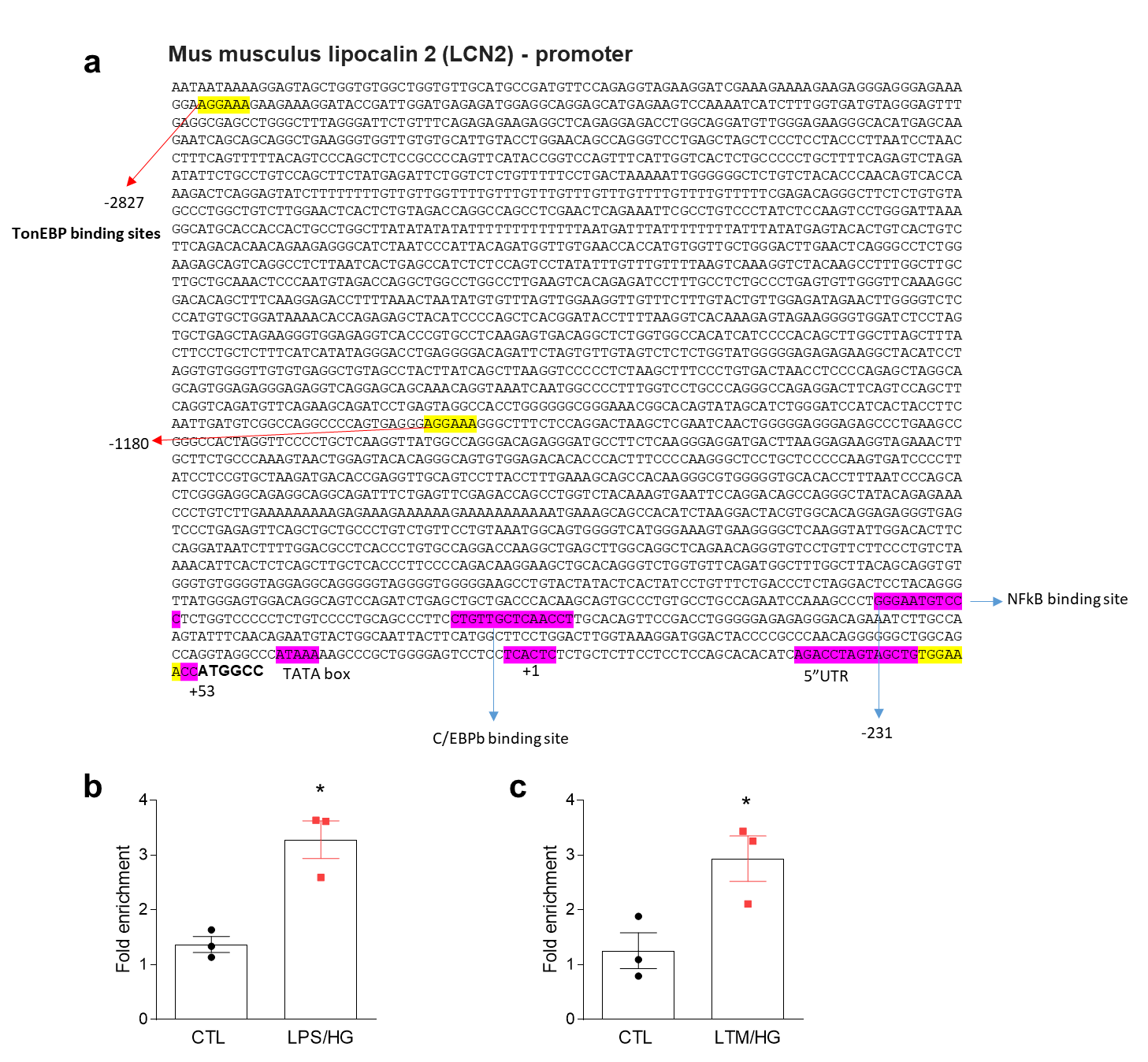
**

**Additional fie 1: Figure S7. The TonEBP binding site in the mouse *LCN2* promoter. (a)** The mouse LCN2 promoter sequence. TonEBP binding consensus sequences at positions −2827 and −1180 in the mouse LCN2 promoter region. **(b** and **c)** ChIP assay for NF-κBp65 at the NF-κB binding site in the *LCN2* promoter LPS/HG-treated RAW264.7 cells (b) (*t* = 7.796, *p* = 0.016) and LTM/HG-treated HT cells (c) (*t* = 6.654, *p* = 0.022). Data (*n* = 3) are shown as mean ± SEM. The *p*-values representing unpaired *t*-tests are indicated in **b** and **c**. **p* < 0.05 versus CTL.

**Additional fie 1: Table S1. List of primary antibodies**

| Antibody | Company | Catalog No. | Dilution(s) | Applications | Source |
| --- | --- | --- | --- | --- | --- |
| LCN2 | R&D | AF3508 | 1:1,000, 1:200 | WB, IF | Goat |
| LCN2 | abcam | ab70287 | 1:200 | IF | Rat |
| NeuN | millipore | MAB377 | 1:500 | IF | Mouse |
| TonEBP | Dr. Kwon |  | 1:3,000, 1:500 | WB, IHC | Rabbit |
| TonEBP | Santa Cruz | sc-5501 | 1:1,000, 1:200 | WB, IF | Goat |
| TonEBP | abcepta | AP74036 | 1:100 | IF | Rabbit |
| GFAP | Sigma | G3893 | 1:1000, 1:500 | WB, IF | Mouse |
| GFAP | Santa Cruz | sc-6170 | 1:200 | IF | Goat |
| NF-κBp65 | Santa Cruz | sc-8008 | 1:1,000  1:200 | WB, IF | Mouse |
| Insulin | abcam | ab7842 | 1:200 | IF | G. pig |
| F4/80 | Santa Cruz | sc-377009 | 1:50 | IHC, IF | Mouse |
| Ly6G | abcam | ab25377 | 1:100 | IHC, IF | Rat |
| NE | abcam | ab68672 | 1:100 | IHC, IF | Rabbit |
| perilipin1 | abcam | ab61682 | 1:200 | IF | Goat |
| CD68 | Santa Cruz | sc-20060 | 1:200 | IF | Mouse |
| CD11b | Millipore | ox-42 | 1:100 | IF | Mouse |
| RAGE | abcam | ab3611 | 1:1,000 | WB | Rabbit |
| Iba1 | Wako | 019-19741 | 1:200 | IF | Rabbit |
| ZO-1 | Santa Cruz | sc-33725 | 1:200 | IF | Rat |
| HO-1 | Enzo | ADI-SPA-895 | 1:1,000, 1:200 | WB, IF | Rabbit |
| VCAM-1 | abcam | ab134047 | 1:1,000 | WB | Rabbit |
| Collagen Ⅳ | abcam | ab6586 | 1:200 | IF | Rabbit |
| PRXⅡ | Santa Cruz | sc-23967 | 1:1,000 | WB | Goat |
| p84 | abcam | ab487 | 1:3,000 | WB | Mouse |
| α-tubulin | Sigma | T5168 | 1:10,000 | WB | Mouse |
| β-actin | Sigma | A5441 | 1:50,000 | WB | Mouse |

WB, western blot; IF, immunofluorescence; IHC, immunohistochemistry

**Additional fie 1: Table S2. List of qRT-PCR primers**

| Gene name | Primer sequences |
| --- | --- |
| *TNF-α* | Forward 5’ CCAGACCCTCACACTCAGATC 3' |
|  | Reverse 5’ CACTTGGTGGTTTGCTACGAC 3' |
| *IL-1β* | Forward 5’ GATCCACACTCTCCAGCTGCA 3' |
|  | Reverse 5’ TACAAGGAGAGACAAGCAACGACA 3' |
| *Arg1* | Forward 5’ AAAGCTGGTCTGCTGGAAAA 3' |
|  | Reverse 5’ ACAGACCGTGGGTTCTTCAC 3' |
| *mrc2* | Forward 5’ TACAGCTCCACGCTATGGATT 3' |
|  | Reverse 5’ CACTCTCCCAGTTGAGGTACT 3' |
| *TonEBP* | Forward 5’ TACCTCAGTCACCGACAGCAAG 3' |
|  | Reverse 5’ CGACTGTTATCCAGCAAGTCCTCA 3' |
| *LCN2* | Forward 5’ CCAGTTCGCCATGGTATTTT 3' |
|  | Reverse 5’ GGTGGGGACAGAGAAGATGA 3' |
| *NF-κBp65* | Forward 5’ GTCTCCATGCAGCTACGGC 3' |
|  | Reverse 5’ GAAGTTGAGTTTCGGGTAGGC 3' |
| *IL-6* | Forward 5’ AGTTGCCTTCTTGGGACTGA 3' |
|  | Reverse 5’ TCCACGATTTCCCAGAGAAC 3' |
| *IL-10* | Forward 5’ CCAGGGAGATCCTTTGATGA 3' |
|  | Reverse 5’ AACTGGCCACAGTTTTCAGG 3' |
| *GAPDH* | Forward 5’ AAATGGTGAAGGTCGGTGTG 3' |
|  | Reverse 5’ CATGTAGTTGAGGTCAATGAAGG 3' |

**Additional fie 1: Table S3. List of qRT-PCR primer for ChIP assay.**

| Gene name | Primer sequences |
| --- | --- |
| *LCN2 (−2827)* | Forward 5’ GGGAGAAAGGAAGGAAAGAAGAA 3' |
|  | Reverse 5’ GCCTCAAACTCCCTACATCAC 3' |
| *LCN2 (−1180)* | Forward 5’ CGGGAAACGGCACAGTATAG 3' |
|  | Reverse 5’ TCGAGCTTAGTCCTGGAGAA 3' |
| *NF-κBp65* | Forward 5’ TGGGAATGTCCCTCTGGTCC 3' |
|  | Reverse 5’ GGTTTCCACAGCTACTAGGT 3' |
| *GAPDH* | Forward 5’ GGGTTCCTATAAATACGGACTGC 3' |
|  | Reverse 5’ CTGGCACTGCACAAGAAGA 3' |

**Additional fie 1: Table S4. Clinical characteristics of normal subjects (CTL) and type 2 diabetic patients (DM) with or without mild cognitive impairment (MCI).**

|  | CTL (n=28) | DM (n=27) | DM-MCI (n=28) |
| --- | --- | --- | --- |
| Age (years) | 72.46 ± 0.78 | 72.48 ± 0.89 | 75 ± 0.85 |
| BMI (kg/m^2^) | 22.16 ± 0.29 | 25.95 ± 0.48^*^ | 25.44 ± 0.4^*^ |
| HbA1C (%) | 5.32 ± 0.03 | 7.3 ± 0.19^*^ | 7.34 ± 0.2^*,†^ |
| Education | 13.89 ± 0.8 | 13.59 ± 0.85 | 14 ± 0.76 |
| Cholesterol (mg/dL) | 188.21 ± 6.31 | 168.04 ± 7.52 | 173.29 ± 7.77 |
| Triglyceride (mg/dL) | 99.54 ± 11.78 | 115.41 ± 10.82 | 138.71 ± 13.66 |
| AST (IU/L) | 21.85 ± 0.97 | 22.8 ± 1.86 | 24.9 ± 1.61 |
| ALT (IU/L) | 15.59 ± 1.3 | 20.9 ± 1.88 | 25.88 ± 2.38^*^ |
| BP systolic (mmHg) | 122.54 ± 2.47 | 128.04 ± 3.07 | 132.07 ± 4.61 |
| BP diastolic (mmHg) | 71.54 ± 1.66 | 70.96 ± 2.03 | 73.86 ± 2.67 |
| LDL cholesterol (mg/dL) | 119.2 ± 5.04 | 99.72 ± 6.85 | 108.61 ± 7.15 |
| HDL cholesterol (mg/dL) | 55.08 ± 2.58 | 49.38 ±2.83 | 47.69 ± 2.58 |
| SVLT-Delayed recall | 6.07 ± 0.37 | 5.33 ± 0.4 | 3 ± 0.3^*,†^ |
| K-MMSE score | 28.43 ± 0.25 | 28.11 ± 0.27 | 25.96 ± 0.37^*,†^ |

Data are shown as mean ± SEM. **p* < 0.05 versus CTL. ^†^*p* < 0.05 versus DM. The indicated *p*-values represent a one-way ANOVA followed by Tukey’s post-hoc test.
